# Postnatal Development of the Perirhinal and Parahippocampal Cortices: A Stereological Study in Macaque Monkeys

**DOI:** 10.1002/cne.70130

**Published:** 2026-01-21

**Authors:** Justine Villard, Loïc J. Chareyron, Pamela Banta Lavenex, David G. Amaral, Pierre Lavenex

**Affiliations:** ^1^ Laboratory of Brain and Cognitive Development, Institute of Psychology University of Lausanne Lausanne Switzerland; ^2^ Cognitive Neuroscience and Neuropsychiatry, Developmental Neurosciences University College London Great Ormond Street Institute of Child Health London UK; ^3^ Faculty of Psychology UniDistance Suisse Brig Switzerland; ^4^ MIND Institute and Department of Psychiatry and Behavioral Sciences University of California at Davis Davis California USA; ^5^ California National Primate Research Center University of California at Davis Davis California USA

**Keywords:** episodic memory, hippocampal formation, infantile amnesia, medial temporal lobe, object memory, path integration, perception, spatial memory

## Abstract

The perirhinal and parahippocampal cortices are two prominent structures of the medial temporal lobe that play essential roles in memory and perceptual processes. In humans, major changes in memory capacities occur within the first 7 years of life, but the neurobiological substrates underlying these changes have long been hypothetical. Previous studies have shown that distinct regions, layers, and cells of the hippocampal formation, including the entorhinal cortex, exhibit different profiles of structural and molecular development. Here, to further understand the postnatal maturation of the medial temporal lobe, we implemented stereological techniques to characterize the structural development of the perirhinal and parahippocampal cortices in macaque monkeys. We found distinct, age‐related differences in volume, neuronal soma size, and neuron number in different layers and subdivisions. Volumetric data indicated a late maturation of areas 36r and 36c compared to areas 35, TF, and TH. There was also an earlier maturation of the superficial layers compared to the deep layers in areas 36r and 36c. We observed a transient increase in neuronal soma size at 6 months of age in several subdivisions. Additionally, we found a decrease in neuron numbers in both the perirhinal and parahippocampal cortices, but particularly in area 35 and layer III of area TF between birth and 6 months. These findings are consistent with the differential maturation of the rostral and caudal entorhinal cortex, which are interconnected with the perirhinal and parahippocampal cortices, respectively. Altogether, they support the theory that the differential maturation of distinct hippocampal circuits underlies the emergence of specific “hippocampus‐dependent” memory processes.

## Graphical abstract

Volumetric data reveal a late maturation of areas 36r and 36c compared to areas 35, TF, and TH, as well as a tendency for superficial layers in areas 36r and 36c to mature earlier than the deep layers. Neuronal soma size exhibits a transient increase at 6 months of age in most subdivisions. Area 35 and layer III of area TF exhibit a decrease in neuron numbers.

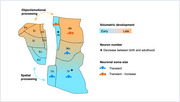

## 1 Introduction

The perirhinal and parahippocampal cortices play essential roles in declarative and spatial memory functions (Squire and Zola 1996; Suzuki and Naya 2014; Zola‐Morgan et al. 1989). They provide the major sources of cortical input to the hippocampal formation, particularly the entorhinal cortex (Insausti et al. 1987; Suzuki and Amaral 1994b), and constitute the main conduit for information between various cortical areas and the medial temporal lobe memory system (Lavenex and Amaral 2000). The most distinctive feature of these two cortices is their contribution to different functional circuits, each conveying specific types of information to the hippocampal formation in support of declarative and spatial memory function. The perirhinal cortex processes mainly information about visual objects and emotional information, while the parahippocampal cortex mainly processes visuospatial information (Stefanacci et al. 1996; Suzuki and Amaral 1994a, 1994b).

In humans, major changes in declarative memory capacities occur within the first 7 years of life (Bauer 2006; Richmond and Nelson 2007), but the neurobiological substrates underlying these changes have long remained hypothetical (Lavenex and Banta Lavenex 2013; Lavenex et al. 2007a). Interestingly, previous studies carried out in monkeys have shown that the hippocampal formation, including the entorhinal cortex, undergoes substantial structural and molecular changes during early postnatal life (Favre et al. 2012a, 2012b; Jabès et al. 2010, 2011; Lavenex et al. 2011; Piguet et al. 2020). In particular, these studies revealed a differential maturation of distinct entorhinal–hippocampal circuits involved in the processing of different types of information, which are thought to underlie the emergence of different “hippocampus‐dependent” memory processes during the first years of life (Lavenex et al. 2024; Lavenex and Banta Lavenex 2013). Given their pivotal role in the medial temporal lobe memory system, it is essential to investigate the postnatal development of the perirhinal and parahippocampal cortices to further evaluate the maturation of distinct medial temporal lobe circuits and better understand the neural substrates underlying the emergence of different types of memories.

### 1.1 Functional Organization of the Monkey Perirhinal and Parahippocampal Cortices

The monkey perirhinal cortex (areas 35, 36r, and 36c) and parahippocampal cortex (areas TH and TF) are characterized by their specific interconnections with a variety of unimodal and polymodal association cortices in the temporal, frontal, and parietal lobes, as well as their close interaction with adjacent structures in the medial temporal lobe, in particular the entorhinal cortex (Amaral and Lavenex 2007; Amaral et al. 2024). The detailed organization of these circuits has been described previously (Lavenex and Amaral 2000; Suzuki and Amaral 1994a, 1994b, 2003; Suzuki and Naya 2014; Villard et al. 2024) and will thus only be briefly summarized here.

The vast majority of cortical inputs toward the monkey perirhinal and parahippocampal cortices comes from visual areas TE, TEO, and V4 (Suzuki and Amaral 1994a), but visual object information from area TE and rostral TEO reaches predominantly the perirhinal cortex, and visuospatial information from area V4, caudal TEO, the retrosplenial, and the posterior parietal cortices targets more specifically the parahippocampal cortex. Projections from the monkey perirhinal and parahippocampal cortices to the entorhinal cortex originate predominantly in layer III and terminate in the superficial layers I, II, and III (Suzuki and Amaral 1994b). Consistent with the view that they contribute to different functional circuits, projections from the perirhinal cortex terminate mainly in the rostral portion of the entorhinal cortex, whereas projections from the parahippocampal cortex terminate mainly in the caudal portion of the entorhinal cortex. Feedback projections from the entorhinal cortex to the perirhinal and parahippocampal cortices follow the same topographical organization as the feedforward projections (Suzuki and Amaral 1994b). In addition, both the perirhinal and parahippocampal cortices send projections to the CA1 region of the hippocampus and the subiculum, but the parahippocampal cortex also sends projections to the presubiculum (Suzuki and Amaral 1990; Van Hoesen et al. 1979). Finally, in contrast to the parahippocampal cortex, the perirhinal cortex shares strong connections with the lateral, basal, and accessory basal nuclei of the amygdala (Stefanacci et al. 1996), suggesting a stronger contribution to the emotional regulation of memory.

Projections from the monkey perirhinal and parahippocampal cortices toward other cortical areas originate from their deep layers and correspond to feedback‐type projections typically observed in cortical areas (Felleman and Van Essen 1991; Lavenex et al. 2002). The parahippocampal cortex shares largely reciprocal connections with other cortical areas, while the perirhinal cortex shares more asymmetric connections (Lavenex et al. 2002), suggesting that the type of information they process necessitates different types of feedback projections to the cortex.

### 1.2 Postnatal Development of Distinct Entorhinal–Hippocampal Circuits in Monkeys

In monkeys, changes in volume and neuronal soma size of distinct hippocampal structures reveal that the parasubiculum, subiculum, CA2, and CA1 exhibit an early maturation before 6 months of age, whereas CA3 and the dentate gyrus exhibit a later structural maturation continuing beyond the first postnatal year (Jabès et al. 2011). In addition, the presubiculum exhibits an early maturation followed by a late reduction of volume and neuronal soma size. As evidence of the strength of quantitative neuroanatomical data, changes in gene‐expression patterns are consistent with the early volumetric maturation of CA1 between birth and 6 months of age, and the late maturation of CA3 between 1 year of age and adulthood (Favre et al. 2012a, 2012b; Lavenex et al. 2011). In addition to a late volumetric development, the dentate gyrus exhibits a protracted period of neuron addition accompanied by a late maturation of the granule cell population (Jabès et al. 2010). No other hippocampal fields exhibit developmental changes in neuron numbers.

A similar analysis of the monkey entorhinal cortex further revealed that its distinct layers and subdivisions exhibit different developmental profiles (Piguet et al. 2020). Volumetric measurements showed a rostrocaudal gradient of maturation with caudal subdivisions exhibiting an early maturation between birth and 6 months of age, and rostral subdivisions exhibiting a late maturation continuing beyond the first postnatal year. The early maturation of the caudal entorhinal cortex, together with the early maturation of hippocampal regions such as CA1, the subiculum, presubiculum, and parasubiculum, suggests an early development of hippocampal circuits involved in spatial processing. The early maturation of these circuits has been proposed to contribute to the early emergence of path integration and basic allocentric spatial processing (Lavenex et al. 2024; Lavenex and Banta Lavenex 2013). In contrast, the late maturation of circuits including the rostral entorhinal cortex, CA3, and the dentate gyrus has been proposed to contribute to the increased precision of allocentric spatial representations and the temporal integration of individual items into episodic memories (Lavenex et al. 2024; Piguet et al. 2020). In addition, the superficial layers of the entorhinal cortex exhibit an overall earlier volumetric maturation than the deep layers, suggesting that cortical inputs can reach and be processed within hippocampal circuits relatively early, but that hippocampal outputs might be directed mainly toward subcortical structures at early ages, and only reach cortical areas at later stages of postnatal development. Finally, although the entorhinal cortex does not exhibit developmental changes in neuron numbers, changes in neuronal soma size are observed across development. In most subdivisions and layers, neuronal soma size typically increases between birth and 6 months of age and then decreases from 6 months to adulthood, with the exceptions of layer III of the caudal areas Elc, Ec, and Ecl in which soma size decreases during postnatal development, and layer II of areas Ec and Ecl in which there is no developmental change in neuronal soma size. Interestingly, these exceptions are consistent with the early maturation of the parasubiculum and the regressive events in the presubiculum, with which the layers II and III of the caudal entorhinal cortex are connected, respectively.

### 1.3 Postnatal Development of the Perirhinal and Parahippocampal Cortices

To our knowledge, there has been no systematic investigation of the postnatal structural development of the perirhinal and parahippocampal cortices that includes neuron number, soma size, and volumes of their different layers and subdivisions. In rats, a morphological analysis and classification of Golgi‐stained perirhinal neurons showed variations in the number and soma size of different cell types across early postnatal development (Furtak et al. 2007). However, the unclear mechanisms by which neurons become impregnated with Golgi staining makes it difficult to extrapolate those findings to the entire neuronal population (Lavenex 2009). Tract‐tracing studies have revealed interesting developmental changes in the connectivity of the perirhinal and parahippocampal cortices. In rats, projections from the postrhinal cortex to the medial entorhinal cortex (homologous to the parahippocampal cortex and caudal entorhinal cortex in primates) exhibit an earlier maturation than projections to the lateral entorhinal cortex (homologous to the rostral entorhinal cortex in primates; Lagartos‐Donate et al. 2022). Providing normative data on the structural development of the perirhinal and parahippocampal cortices is thus critical to better understand the maturation of the primate medial temporal lobe memory system.

### 1.4 Aim of the Study

The aim of this study was to provide normative data on the structural development of the rhesus monkey (*Macaca mulatta*) perirhinal and parahippocampal cortices during early postnatal life. We implemented design‐based stereological techniques to provide estimates of neuron number, neuronal soma size, and volume of the different layers and subdivisions of the perirhinal and parahippocampal cortices of macaque monkeys at 1 day, 6 months, 1 year, and 5–9 years of age. Consistent with previous findings on the structural development of the hippocampal formation (Jabès et al. 2010, 2011), including the entorhinal cortex (Piguet et al. 2020), our results further show the differential maturation of distinct medial temporal lobe circuits. This study provides fundamental information on the normal development of the primate medial temporal lobe memory system and defines critical periods of maturation that might be sensitive to perturbation and contribute to developmental disorders.

## 2 Materials and Methods

### 2.1 Experimental Animals

Sixteen rhesus monkeys, *Macaca mulatta*; four 1‐day‐olds (two M, two F), four 6‐month‐olds (two M, two F), four 1‐year‐olds (two M, two F), and four adults (5.3, 9.4 [M], 7.7 and 9.3 [F] years of age), were used for this study. Monkeys were born from multiparous mothers and raised at the California National Primate Research Center (RRID:SCR_000696). They were maternally reared in 2000 m^2^ outdoor enclosures and lived in large social groups until they were killed. These monkeys were some of the same animals used in quantitative studies of the monkey hippocampal formation (Jabès et al. 2010, 2011), amygdala (Chareyron et al. 2011, 2012, 2016, 2021), entorhinal cortex (Piguet et al. 2018, 2020; Villard et al. 2023), and perirhinal cortex (Villard et al. 2023; 2024). All experimental procedures were approved by the Institutional Animal Care and Use Committee of the University of California, Davis and were in accordance with the National Institutes of Health guidelines for the use of animals in research.

### 2.2 Histological Processing

#### 2.2.1 Brain Acquisition

Monkeys were deeply anesthetized with an intravenous injection of sodium pentobarbital (50 mg/kg iv; Fatal‐Plus; Vortech Pharmaceuticals, Dearborn, MI, USA) and perfused transcardially with 1% and then 4% paraformaldehyde in 0.1 M phosphate buffer (PB; pH 7.4) following protocols previously described (Lavenex et al. 2009). Coronal sections were cut with a freezing microtome in six series at 30‐µm thick, and one series at 60‐µm (Microm HM 450, Microm International GmbH, Walldorf, Baden‐Württemberg, Germany). The 60‐µm sections were collected in 10% formaldehyde solution in 0.1 M PB (pH 7.4) and postfixed at 4°C for 4 weeks prior to Nissl staining with thionin. All other series were collected in tissue collection solution (TCS) and kept at −70°C until further processing.

#### 2.2.2 Nissl Staining With Thionin

The procedure for Nissl‐stained sections followed our standard laboratory protocol described previously (Lavenex et al. 2009). Sections were taken out of the 10% formaldehyde solution, thoroughly washed 2 × 2 h in 0.1 M PB, mounted on gelatin‐coated slides from filtered 0.05 M PB (pH 7.4), and air‐dried overnight at 37°C. Sections were then defatted 2 × 2 h in a mixture of chloroform/ethanol (1:1, vol.), and rinsed 2 × 2 min in 100% ethanol, 2 min in 95% ethanol, and air‐dried overnight at 37°C. Sections were then rehydrated through a graded series of ethanol, 2 min in 95% ethanol, 2 min in 70% ethanol, 2 min in 50% ethanol, dipped in two separate baths of dH_2_O, and stained 20 s in a 0.25% thionin solution (Fisher Scientific, Waltham, MA, USA, cat# T‐409), dipped in two separate baths of dH_2_O, 4 min in 50% ethanol, 4 min in 70% ethanol, 4 min in 95% ethanol + glacial acetic acid (1 drop per 100 mL of ethanol), 4 min in 95% ethanol, 2 × 4 min in 100% ethanol, 3 × 4 min in xylene, and coverslipped with the mounting medium DPX (BDH Laboratories, Poole, UK).

### 2.3 Stereological Analyses

#### 2.3.1 Neuron Number

**FIGURE 1:**
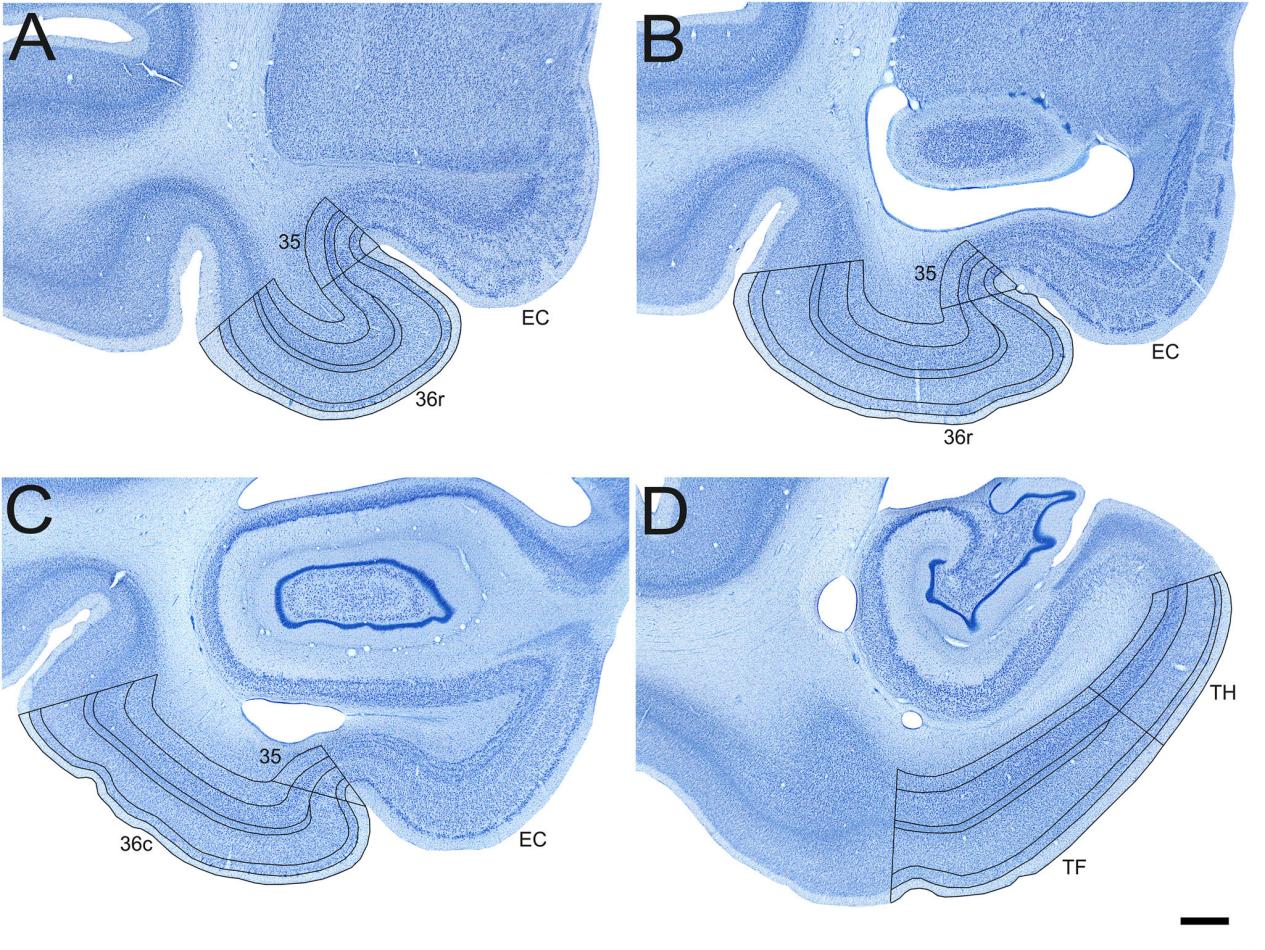
Low‐magnification photomicrographs of Nissl‐stained coronal sections through the adult rhesus monkey perirhinal and parahippocampal cortices. Scale bar = 1 mm, applies to all panels. Panels (A)–(D) go from rostral to caudal. See the main text for details.

**FIGURE 2:**
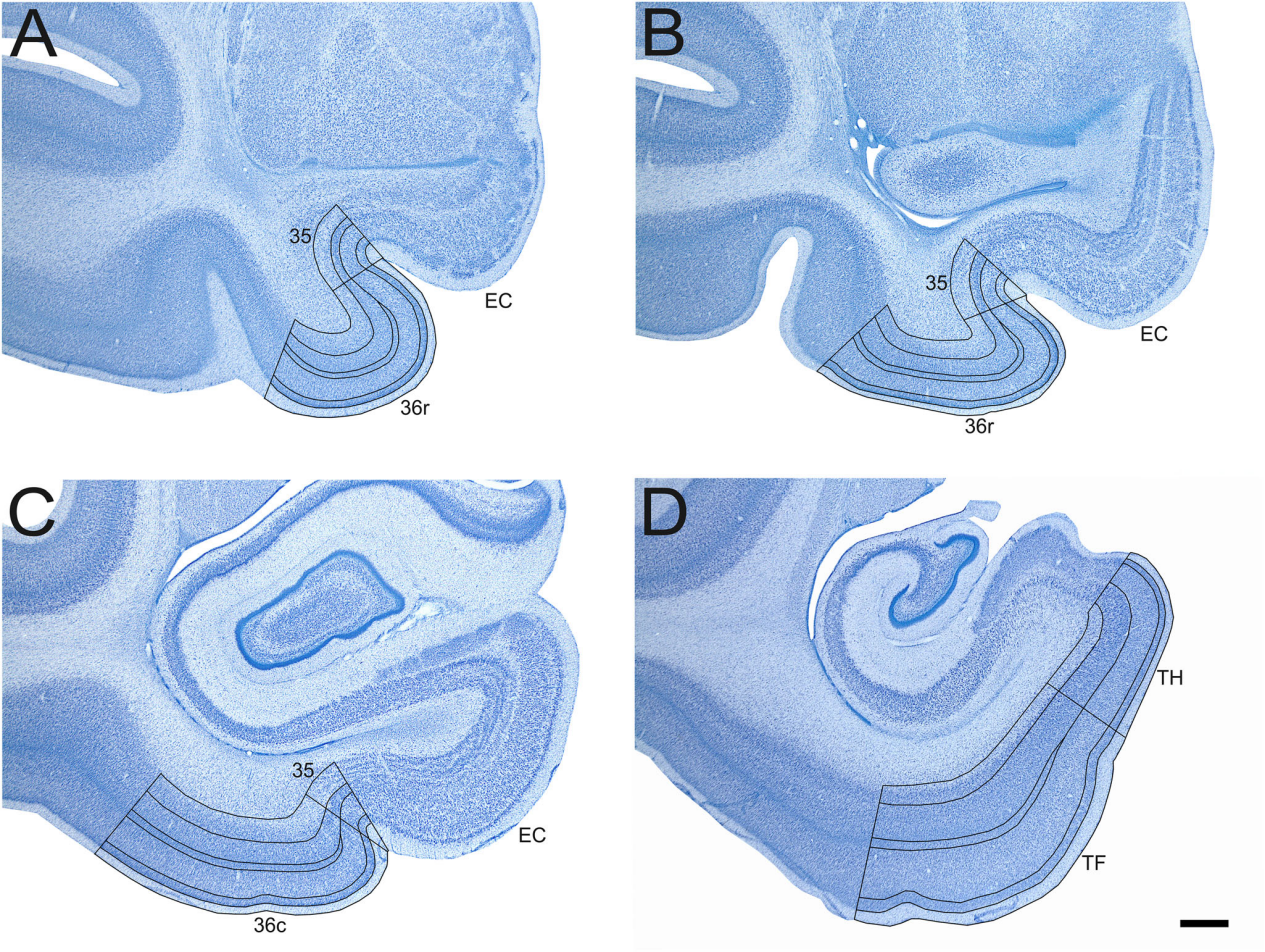
Low‐magnification photomicrographs of Nissl‐stained coronal sections through the neonate rhesus monkey perirhinal and parahippocampal cortices. Scale bar = 1 mm, applies to all panels. Panels (A)–(D) go from rostral to caudal. See the main text for details.

**FIGURE 3:**
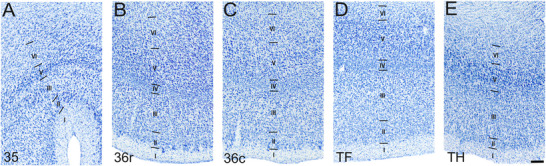
High‐magnification photomicrographs of Nissl‐stained coronal sections through the adult rhesus monkey perirhinal and parahippocampal cortices. Scale bar = 250 µm, applies to all panels. (A) Area 35. (B) Area 36r. (C) Area 36c. (D) Area TF. (E) Area TH. See the main text for details.

**FIGURE 4:**
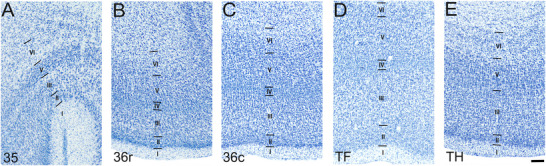
High‐magnification photomicrographs of Nissl‐stained coronal sections through the neonate rhesus monkey perirhinal and parahippocampal cortices. Scale bar = 250 µm, applies to all panels. (A) Area 35. (B) Area 36r. (C) Area 36c. (D) Area TF. (E) Area TH. See the main text for details.

The number of neurons in the different layers (II, III, IV, V, VI) of the distinct subdivisions of the perirhinal (35, 36r, 36c) and parahippocampal (TH, TF) cortices was determined using the optical fractionator method on the Nissl‐stained sections cut at 60 µm (West et al. 1991). Neuron number was not estimated in layer I, which contains very few neurons that are not considered in standard models of hippocampal–cortical interactions (Lavenex and Amaral 2000; Lavenex et al. 2024). Each area and layer were delineated according to previously described cytoarchitectural criteria of the monkey perirhinal and parahippocampal cortices (Figures 1, 2, 3, 4; Suzuki and Amaral 1994a). We estimated neuron numbers using the following formula: *N* = ∑*Q* × 1/ssf × 1/asf × 1/tsf (ssf: section sampling fraction; asf: area sampling fraction; tsf: thickness sampling fraction). This design‐based method enables an estimation of the absolute number of neurons that is independent of the volume. From 12 to 16 sections (960 µm apart) per animal were used for these analyses, with the first section selected randomly within the first two sections through which area 35 was clearly identifiable around the rhinal sulcus, corresponding approximately to the most rostral portion of the entorhinal cortex visible on coronal sections. Note that this rostral border is a little more restrictive than in previous studies (Lavenex et al. 2004; Suzuki and Amaral 1994a), but was considered more reliable for the stereological analyses with respect to the plane of cutting. Neuron number was estimated in the right hemisphere for half of the animals and in the left hemisphere for the other half. We used a 100× Plan Fluor oil objective (N.A. 1.30) on a Nikon Eclipse 80i microscope (Nikon Instruments Inc., Melville, NY, USA) linked to PC‐based StereoInvestigator 2019 (RRID:SCR_002526; MBF Bioscience, Williston, VT, USA). Sampling schemes were established to obtain individual estimates of neuron number with coefficients of error (CE) around 0.15 using the following equation:

$$\mathrm{CE}=\sqrt{{\left(\frac{\frac{3\left(A-S^{2}\right)-4B+C}{12}+S^{2}}{\Sigma Q_{i}}\right)}^{2}+{\left(\frac{\mathrm{stdev}\;\mathrm{thickness}}{\mathrm{mean}\;\mathrm{thickness}}\right)}^{2}}$$

where $S^{2}$ is the variance due to noise, formerly the nugget, and

$$A=\Sigma Q_{i}^{2}\;B=\Sigma Q_{i}Q_{i+1}\;C=\Sigma \;Q_{i}Q_{i+2}$$

The first term of the equation corresponds to the coefficient of variation of neuron number typically used in stereological studies (for further information, see https://www.stereology.info/some‐ce‐theory/; Basler et al. 2017; Gundersen and Jensen 1987; Gundersen et al. 1999) to which a CE for the variation of section thickness is added. In general, the latter is not included in the basic calculations and inevitably leads to overall higher theoretical CEs (around 0.15 in the present study), but it is an important factor to consider since the estimates of neuron number are highly dependent on the section thickness (for more information, see Peruzzi 2012). Note that empirical CEs based on true replicates of neuron number estimates are typically lower than the theoretical CEs calculated from these formulas (data not shown).

The stereological sampling schemes are presented in Table 1. Neurons were counted when their nucleolus (or nucleus if there were more than one nucleolus) came into focus within the counting frame, as it was moved through a known distance of the section thickness. We identified neurons based on morphological criteria identifiable in Nissl preparations, as previously described (Chareyron et al. 2016, 2012; García‐Cabezas et al. 2016; Piguet et al. 2018). García‐Cabezas et al. (2016) provided a particularly well‐illustrated and detailed description of the cytology of Nissl‐stained neurons and glial cells. Briefly, neurons are darkly stained and comprise a single large nucleolus. Astrocytes are relatively smaller in size and exhibit pale staining of the nucleus. Immature neurons, as described in Chareyron et al. (2016), are present in the monkey areas 35 and 36 of the perirhinal cortex and were quantified for area 36 with the same stereological parameters used previously (Villard et al. 2023). They are relatively smaller than astrocytes, have a round to slightly oval, hyperchromatic nuclei containing distinguishable nucleoli. Oligodendrocytes are just as small as immature neurons and contain round, darkly stained nuclei that are densely packed with chromatin. Microglia have the smallest nucleus, dark staining, and an irregular shape that is often rod‐like, oval, or bent (Villard et al. 2024).

**TABLE 1:** Parameters used for the stereological analysis of the rhesus monkey perirhinal and parahippocampal cortices.

Area	Layer	Number of sections	Distance between sections (µm)	Scan grid (µm)^a^	Counting frame (µm)	Disector height (µm)	Guard zones (µm)	Average section thickness^b^	Number of neurons counted (range)	Number of disectors (range)	Coefficients of error (CE)
35	II	6–8	960	100 × 100	40 × 40	5	2	13.07 (9.5–15.6)	216 (130–355)	129 (100–166)	0.13 (0.10–0.16)
	III	6–8	960	150 × 150	40 × 40	5	2	13.37 (9.4–15.8)	165 (100–280)	109 (75–152)	0.13 (0.10–0.18)
	V	6–8	960	150 × 150	40 × 40	5	2	13.30 (9.2–17.1)	165 (102–268)	98 (71–124)	0.13 (0.10–0.20)
	VI	6–8	960	200 × 200	40 × 40	5	2	12.63 (8.8–16.5)	174 (115–265)	113 (93–143)	0.13 (0.10–0.18)
36r	II	4–6	960	200 × 200	20 × 20	5	2	11.44 (8.4–13.2)	202 (128–361)	207 (125–328)	0.14 (0.11–0.17)
	III	4–6	960	400 × 400	30 × 30	5	2	12.39 (8.7–15.0)	161 (109–286)	114 (62–190)	0.14 (0.10–0.16)
	IV	4–6	960	200 × 200	20 × 20	5	2	12.58 (8.8–15.1)	121 (78–221)	96 (68–157)	0.14 (0.11–0.19)
	V	4–6	960	300 × 300	30 × 30	5	2	12.52 (9.1–15.3)	178 (106–308)	115 (77–188)	0.14 (0.10–0.17)
	VI	4–6	960	250 × 250	30 × 30	5	2	12.28 (8.4–15.2)	147 (80–299)	115 (72–203)	0.15 (0.11–0.17)
36c	II	3–5	960	200 × 200	20 × 20	5	2	11.81 (8.0–14.1)	123 (71–230)	126 (72–215)	0.18 (0.14–0.25)
	III	3–5	960	300 × 300	30 × 30	5	2	12.78 (8.0–15.6)	174 (98–324)	122 (75–167)	0.17 (0.13–0.21)
	IV	3–5	960	150 × 150	20 × 20	5	2	13.05 (8.3–15.8)	127 (60–227)	98 (63–177)	0.18 (0.15–0.27)
	V	3–5	960	230 × 230	30 × 30	5	2	13.00 (8.4–16.0)	191 (99–370)	127 (74–205)	0.17 (0.13–0.24)
	VI	3–5	960	200 × 200	30 × 30	5	2	12.85 (8.2–16.3)	152 (83–329)	122 (77–184)	0.17 (0.13–0.26)
TH	II	3–5	960	170 × 170	30 × 30	5	2	12.28 (8.7–15.2)	226 (157–382)	104 (66–132)	0.15 (0.09–0.26)
	III	3–5	960	270 × 270	30 × 30	5	2	12.87 (8.6–16.0)	182 (128–194)	104 (70–132)	0.16 (0.10–0.25)
	V	3–5	960	230 × 230	30 × 30	5	2	13.14 (8.5–16.1)	148 (98–276)	83 (70–104)	0.15 (0.11–0.21)
	VI	3–5	960	150 × 150	30 × 30	5	2	12.89 (8.1–15.7)	137 (82–225)	106 (80–139)	0.16 (0.10–0.21)
TF	II	6–9	960	320 × 320	20 × 20	5	2	12.46 (8.5–15.8)	129 (79–277)	108 (70–159)	0.15 (0.11–0.22)
	III	6–9	960	550 × 550	30 × 30	5	2	13.42 (8.5–16.5)	155 (93–349)	94 (66–112)	0.15 (0.11–0.20)
	IV	6–9	960	220 × 220	20 × 20	5	2	13.63 (8.7–17.0)	168 (109–266)	121 (77–183)	0.13 (0.10–0.16)
	V	6–9	960	430 × 430	30 × 30	5	2	13.43 (8.2–17.0)	175 (117–323)	97 (73–139)	0.12 (0.10,0.18)
	VI	6–9	960	330 × 330	30 × 30	5	2	13.17 (8.2–16.9)	132 (85–259)	96 (66–124)	0.13 (0.10–0.18)
36 (immature neurons)	II	7–10	960	250 × 250	40 × 40	5	2	11.87 (8.9–13.8)	264 (111–457)	231 (147–372)	0.12 (0.09–0.19)

^a^ Scan grid was placed in a random orientation.

^b^ Section thickness was measured at every other counting site.

#### 2.3.2 Volume Estimates

We estimated the volume of individual layers in each subdivision of the monkey perirhinal and parahippocampal cortex based on the outline tracings performed with StereoInvestigator 2019 for the estimation of neuron numbers (Figures 1 and 2). We used the section cutting thickness (60 µm) to determine the distance between sampled sections, which was then multiplied by the total surface area delineated for neuron counts to calculate the volume.

#### 2.3.3 Neuronal Soma Size

The volume of the soma of every neuron counted during the optical fractionator analysis was determined using the nucleator method (Gundersen 1988), as described previously (Villard et al. 2024). Note that the nucleator method provides accurate estimates of neuron size when isotropic‐uniform‐random sectioning of brain structures is employed. In our study all brains were cut in the coronal plane. Estimates of cell size might therefore be impacted by the nonrandom orientation of neurons in the different layers and subdivisions of the perirhinal and parahippocampal cortices, which could lead to an overestimation or underestimation of cell size in any given structure.

### 2.4 Statistical Analyses

We used SPSS 29 (IBM statistics; RRID:SCR_002865) to perform general linear model (GLM) analyses with age as a factor, and regions and layers as repeated measures, to compare the postnatal development of the distinct subdivisions and layers of the perirhinal and parahippocampal cortices. Degrees of freedom were adjusted following the Greenhouse–Geisser method when the Mauchly's test of sphericity for repeated measures was significant. We report effect size with *η* ^2^_p_ (partial eta squared). We performed post hoc analyses with the Fisher‐LSD test when the *F* ratio was significant, thus controlling for Type I error rate (Carmer and Swanson 1973). Significance level was set at a two‐tailed *p* value < 0.05 for GLM analyses and post hoc tests, unless specified otherwise for some comparisons between individual age groups. Since the general structural differences between the distinct subdivisions and layers of the adult monkey perirhinal and parahippocampal cortices has been the focus of a previous study (Villard et al. 2024), the statistical analyses of the current study mainly focused on age group differences. Developmental profiles were defined semiquantitatively, based on both the results of the statistical tests and the general trends of age group differences. Given that these developmental profiles corresponded to those previously described in the monkey entorhinal cortex (Piguet et al. 2020), we used the same definitions to describe the developmental profile of each layer and subdivision of the perirhinal and parahippocampal cortices. Volumetric developmental profiles: (a) Very early maturation: newborn = 6 months = 1 year = 5–9 years. (b) Early maturation: newborn < 6 months = 1 year = 5–9 years. (c) Two‐step maturation: newborn < 6 months = 1 year < 5–9 years. (d) Gradual maturation: newborn < 5–9 years. (e) Late maturation: newborn = 6 months = 1 year < 5–9 years. Developmental profiles of neuronal soma size: (a) No change: newborn = 6 months = 1 year = 5–9 years. (b) Transient: a transient increase at intermediate ages, newborn < 6 months ‐ 1 year > 5–9 years, no difference between newborn and 5–9 years. (c) Transient‐increase: a transient increase at intermediate ages, together with a net increase between birth and adulthood: newborn < 6 months ‐ 1 year > 5–9 years and newborn < 5–9 year. (d) Late decrease: newborn = 6 months = 1 year > 5–9 years. (e) Transient‐decrease: a transient increase at intermediate ages, together with a net decrease between birth and adulthood: newborn < 6 months ‐ 1 year > 5–9 years and newborn > 5–9 years. This developmental profile was added to characterize the specific maturation of layer IV, which is absent in the entorhinal cortex. Percentages of adult values are reported on figures to facilitate the comparisons of different developmental patterns.

### 2.5 Photomicrographic Production

Photomicrographs were taken with a Leica DFC420 digital camera on a Leica MZ9.5 stereomicroscope (Leica Microsystems GmbH, 35578, Wetzlar, Germany). Artifacts located outside the sections were removed, and color balance was adjusted in Adobe Photoshop V 24.0.1 (RRID:SCR_014199; Adobe Systems, San Jose, CA, USA) to improve contrast and clarity.

## 3 Results

### 3.1 Areal Volume

**FIGURE 5:**
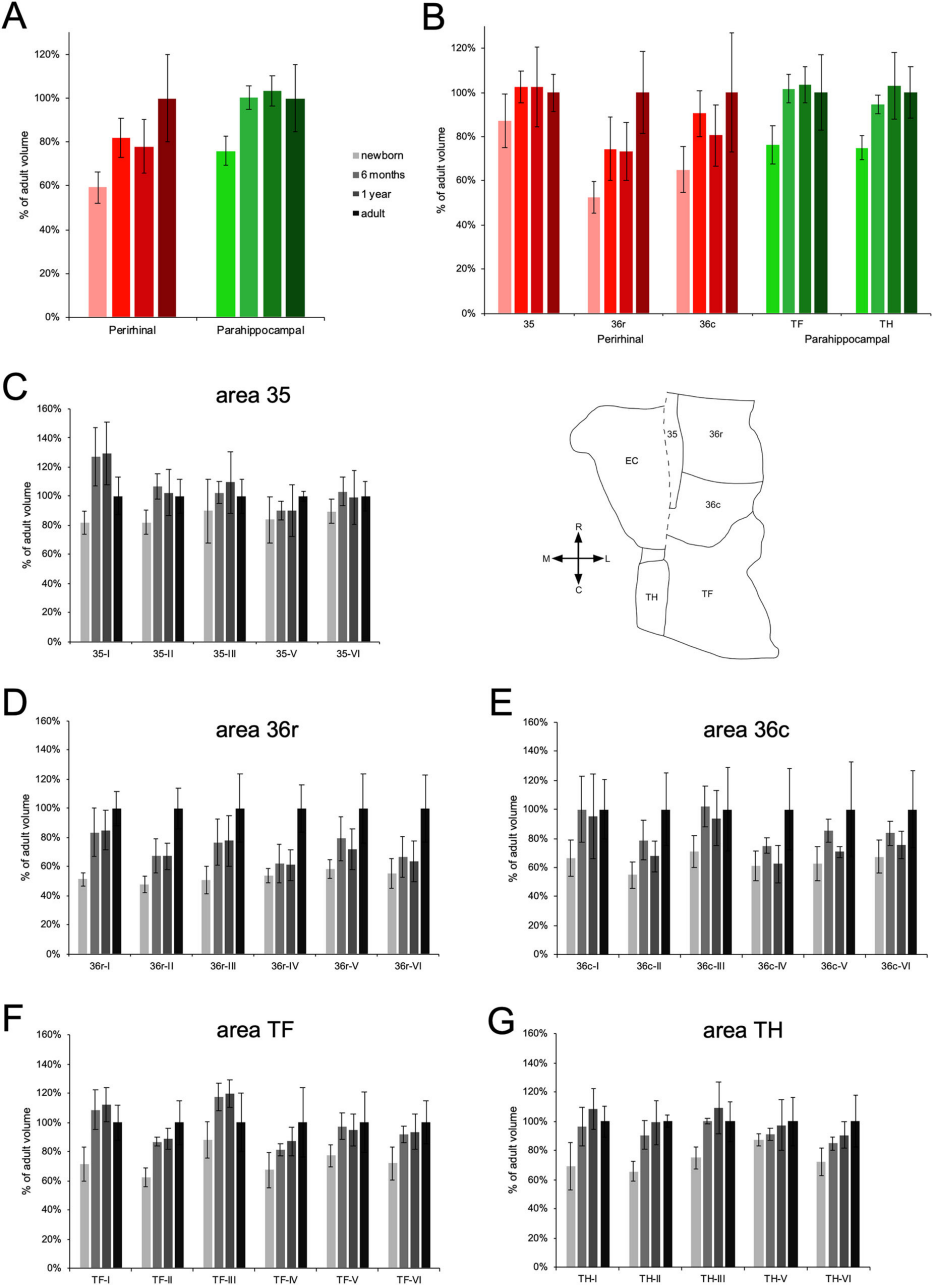
Percentage of the adult volume of the distinct layers and subdivisions of the monkey perirhinal and parahippocampal cortices, at different postnatal ages. (A) Volumes of the perirhinal cortex and parahippocampal cortex. (B) Volumes of individual subdivisions. Volumes of individual layers within each subdivision: (C) area 35; (D) area 36r; (E) area 36c; (F) area TF; (G) area TH. The unfolded map illustrates the relative position of each subdivision. The dashed line indicates the position of the rhinal sulcus.

The volumes of the different layers of the distinct subdivisions of the perirhinal and parahippocampal cortices at different postnatal ages are presented in Tables 2 and 3 and Figure 5. Both the perirhinal cortex (*F*_(3,12)_ = 6.586, *p* = 0.007, η^2^_p_ = 0.662) and the parahippocampal cortex (*F*_(3,12)_ = 7.219, *p* = 0.005, η^2^_p_ = 0.643) exhibited differences in volumes between different postnatal ages (Figure 5A), but the developmental patterns differed between the two cortices (cortex × age group: *F*_(3,12)_ = 3.214, *p* = 0.062, η^2^_p_ = 0.446). The volume of the perirhinal cortex exhibited a two‐step maturational profile, with a first increase between birth and 6 months of age (*p* = 0.030), when it was 82% of its adult volume, and a second increase between 1 year and adulthood (*p* = 0.034). In contrast, the volume of the parahippocampal cortex exhibited an early maturational profile, with a major increase between birth and 6 months of age (*p* = 0.003), when it reached 100% of its adult volume.

**TABLE 2:** Average volume (mm^3^ ± *SD*) of the different layers and subdivisions of the rhesus monkey perirhinal and parahippocampal cortices at four postnatal ages.

Area	Layer	Newborn	6 months	1 year	5–9 years
35	I	0.62 ± 0.06	0.96 ± 0.15	0.97 ± 0.16	0.75 ± 0.10
	II	0.78 ± 0.08	1.01 ± 0.08	0.97 ± 0.15	0.95 ± 0.11
	III	1.69 ± 0.41	1.92 ± 0.14	2.06 ± 0.40	1.88 ± 0.22
	V	1.51 ± 0.29	1.62 ± 0.11	1.62 ± 0.32	1.80 ± 0.07
	VI	3.42 ± 0.32	3.93 ± 0.37	3.78 ± 0.72	3.81 ± 0.39
	All layers	8.02 ± 1.10	9.44 ± 0.66	9.40 ± 1.66	9.19 ± 0.78
36r	I	5.37 ± 0.47	8.74 ± 1.73	8.91 ± 1.41	10.48 ± 1.25
	II	4.73 ± 0.55	6.69 ± 1.17	6.66 ± 0.91	9.91 ± 1.36
	III	11.01 ± 2.04	16.58 ± 3.45	16.81 ± 3.70	21.66 ± 5.06
	IV	2.40 ± 0.23	2.79 ± 0.59	2.73 ± 0.46	4.47 ± 0.72
	V	6.70 ± 0.76	9.09 ± 1.72	8.29 ± 1.61	11.49 ± 2.73
	VI	4.75 ± 0.89	5.75 ± 1.19	5.48 ± 1.18	8.61 ± 1.98
	All layers	34.96 ± 4.72	49.64 ± 9.70	48.88 ± 8.73	66.62 ± 12.33
36c	I	3.34 ± 0.63	5.01 ± 1.14	4.78 ± 1.45	5.02 ± 1.01
	II	2.94 ± 0.49	4.22 ± 0.74	3.63 ± 0.57	5.37 ± 1.34
	III	7.60 ± 1.20	10.95 ± 1.50	10.06 ± 2.05	10.71 ± 3.09
	IV	1.48 ± 0.25	1.82 ± 0.14	1.51 ± 0.31	2.42 ± 0.68
	V	4.65 ± 0.89	6.36 ± 0.58	5.26 ± 0.29	7.44 ± 2.42
	VI	3.48 ± 0.57	4.30 ± 0.45	3.89 ± 0.47	5.16 ± 1.38
	All layers	23.49 ± 3.74	32.66 ± 3.74	29.13 ± 4.99	36.12 ± 9.75
Perirhinal cortex		66.47 ± 7.94	91.74 ± 9.79	87.41 ± 13.89	111.93 ± 22.23
TF	I	7.70 ± 1.27	11.69 ± 1.49	12.10 ± 1.26	10.75 ± 1.30
	II	7.00 ± 0.70	9.73 ± 0.34	10.00 ± 0.80	11.25 ± 1.71
	III	21.33 ± 2.95	28.47 ± 2.20	29.07 ± 2.28	24.23 ± 4.83
	IV	3.85 ± 0.70	4.63 ± 0.24	4.98 ± 0.56	5.71 ± 1.35
	V	13.13 ± 1.27	16.54 ± 1.54	16.14 ± 1.88	17.00 ± 3.53
	VI	7.09 ± 1.09	9.09 ± 0.55	9.25 ± 1.17	9.87 ± 1.46
	All layers	60.10 ± 6.68	80.15 ± 4.98	81.54 ± 6.33	78.81 ± 13.37
TH	I	2.40 ± 0.56	3.34 ± 0.46	3.75 ± 0.48	3.46 ± 0.37
	II	1.72 ± 0.19	2.37 ± 0.26	2.60 ± 0.39	2.62 ± 0.11
	III	5.27 ± 0.54	7.02 ± 0.13	7.67 ± 1.25	7.02 ± 0.95
	V	3.52 ± 0.17	3.68 ± 0.17	3.93 ± 0.70	4.04 ± 0.68
	VI	1.64 ± 0.21	1.92 ± 0.10	2.05 ± 0.21	2.27 ± 0.40
	All layers	14.55 ± 1.06	18.33 ± 0.83	20.00 ± 2.91	19.41 ± 2.22
Parahippocampal cortex		74.65 ± 6.56	98.48 ± 5.39	101.54 ± 6.98	98.22 ± 14.97

**TABLE 3:** Results of the statistical analyses on the volume and attribution of distinct maturation profiles to the different layers and subdivisions of the monkey perirhinal and parahippocampal cortices.

Area	Layer	Profile^a^	*F*	*p*	η^2^_p_	Post hoc comparisons with *p* < 0.05
35	I	Transient	7.455	0.004	0.651	Newborn < 6 months ‐ 1 year > 5–9 years
	II	Early	3.557	0.048	0.471	Newborn < 6 months ‐ 1 year to 5–9 years
	III	Very early	0.937	0.453	0.190	—
	V	Very early	1.172	0.361	0.227	—
	VI	Very early	0.872	0.482	0.179	—
	All layers	Very early	1.472	0.272	0.269	—
36r	I	Early	10.933	< 0.001	0.732	Newborn < 6 months ‐ 1 year to 5–9 years
	II	Two‐step	16.936	< 0.001	0.809	Newborn < 6 months ‐ 1 year < 5–9 years
	III	Two‐step	5.489	0.013	0.578	Newborn < 1 year ‐ 5–9 years
	IV	Late	12.176	< 0.001	0.753	Newborn ‐ 6 months ‐ 1 year < 5–9 years
	V	Late	4.711	0.021	0.541	Newborn ‐ 1 year < 5–9 years
	VI	Late	6.139	0.009	0.605	Newborn ‐ 6 months ‐ 1 year < 5–9 years
	All layers	Two‐step	7.805	0.004	0.661	Newborn < 6 months ‐ 1 year < 5–9 years
36c	I	Early	2.158	0.146	0.350	—
	II	Late	5.818	0.011	0.593	Newborn ‐ 6 months ‐ 1 year < 5–9 years
	III	Early	2.155	0.147	0.350	Newborn < 6 months ‐ 1 year ‐ 5–9 years
	IV	Late	4.802	0.020	0.546	Newborn ‐ 1 year < 5–9 years
	V	Late	3.453	0.051	0.463	Newborn ‐ 1 year < 5–9 years
	VI	Late	3.106	0.067	0.437	Newborn ‐ 1 year < 5–9 years
	All layers	Early	3.150	0.065	0.441	Newborn < 5–9 years
Perirhinal cortex		Two‐step	6.586	0.007	0.622	Newborn < 6 months ‐ 1 year < 5–9 years
TF	I	Early	8.881	0.002	0.689	Newborn < 6 months ‐ 1 year ‐ 5–9 years
	II	Early	12.357	< 0.001	0.755	Newborn < 6 months ‐ 1 year ‐ 5–9 years
	III	Transient	5.098	0.017	0.560	Newborn < 6 months ‐ 1 year
	IV	Gradual	3.549	0.048	0.470	Newborn < 5–9 years
	V	Early	2.462	0.113	0.381	Newborn < 5–9 years
	VI	Early	4.678	0.022	0.539	Newborn < 6 months ‐ 1 year ‐ 5–9 years
	All layers	Early	5.658	0.012	0.586	Newborn < 6 months ‐ 1 year ‐ 5–9 years
TH	I	Early	6.130	0.009	0.605	Newborn < 6 months ‐ 1 year ‐ 5–9 years
	II	Early	10.534	0.001	0.725	Newborn < 6 months ‐ 1 year ‐ 5–9 years
	III	Early	6.113	0.009	0.604	Newborn < 6 months ‐ 1 year ‐ 5–9 years
	V	Very early	0.888	0.475	0.182	—
	VI	Gradual	4.246	0.029	0.515	Newborn < 1 year ‐ 5–9 years
	All layers	Early	6.292	0.008	0.611	Newborn < 6 months ‐ 1 year ‐ 5–9 years
Parahippocampal cortex		Early	7.219	0.005	0.643	Newborn < 6 months ‐ 1 year ‐ 5–9 years

^a^ Profiles were defined semiquantitatively, based on both statistical tests and general trends of age group differences.

Interestingly, in addition to the general developmental profiles found for these two cortices, there were clear differences in the developmental profiles of distinct subdivisions (35, 36r, 36, TF, and TH; Figure 5B; subdivision × age group: *F*_(7.612, 30.447)_ = 4.532, *p* = 0.001, η^2^_p_ = 0.531), which will be further described below.

#### 3.1.1 Perirhinal Cortex

##### 3.1.1.1 Subdivisions

The three subdivisions of the perirhinal cortex exhibited different volumetric developmental profiles (Figure 5B; subdivision × age group: *F*_(6, 24)_ = 6.058, *p* < 0.001, η^2^_p_ = 0.602). The volumes of area 36r (*F*_(3,12)_ = 7.805, *p* = 0.004, η^2^_p_ = 0.661) and area 36c (*F*_(3,12)_ = 3.150, *p* = 0.065, η^2^_p_ = 0.441) differed between age groups, whereas the volume of area 35 did not (*F*_(3,12)_ = 1.472, *p* = 0.272, η^2^_p_ = 0.269). The volume of area 36r exhibited a two‐step maturational profile, first increasing between birth and 6 months of age (*p* = 0.045), to attain 75% of its adult volume, and a second increase between 1 year of age and adulthood (*p* = 0.019). The volume of area 36c exhibited an early maturational profile, with an increase between birth and 6 months of age (*p* = 0.054), when it attained 90% of its adult volume. The volume of area 35 exhibited a very early maturational profile, as its volume at birth was 87% of its adult volume.

##### 3.1.1.2 Layers Within Each Subdivision

In area 35, the distinct layers did not exhibit statistically significant differences in their volumetric developmental profiles (Figure 5C; layer × age group: *F*_(4.847,19.388)_ = 0.923, *p* = 0.485, η^2^_p_ = 0.188). Nevertheless, layer I exhibited a transient increase in volume at 6 months and 1 year of age. In contrast, the other layers exhibited an early (layer II) or very early (layers III–VI) volumetric maturation between birth and 6 months of age, when they were 82%–90% of their adult volume (Tables 2 and 3).

In area 36r, the distinct layers exhibited different volumetric developmental profiles (Figure 5D; layer × age group: *F*_(4.088,16.352)_ = 4.071, *p* = 0.017, η^2^_p_ = 0.504). Layer I (*F*_(3,12)_ = 10.933, *p* < 0.001, η^2^_p_ = 0.732) matured early, with a major increase in volume between birth and 6 months of age (*p* = 0.003), when it attained 83% of its adult volume. Layer II (*F*_(3,12)_ = 16.936, *p* < 0.001, η^2^_p_ = 0.809) and layer III (*F*_(3,12)_ = 5.489, *p* = 0.013, η^2^_p_ = 0.578) exhibited a two‐step maturational profile; a first increase in volume between birth and 6 months of age (layer II, *p* = 0.021; layer III, *p* = 0.056), when they were 67% and 77% of their adult volume, respectively; and a second increase between 1 year of age and adulthood (layer II, *p* < 0.001; layer III, *p* = 0.090). Layer IV (*F*_(3,12)_ = 12.176, *p* < 0.001, η^2^_p_ = 0.753), layer V (*F*_(3,12)_ = 4.711, *p* = 0.021, η^2^_p_ = 0.541), and layer VI (*F*_(3,12)_ = 6.139, *p* = 0.009, η^2^_p_ = 0.605) of area 36r exhibited a late maturational profile with an increase in volume between 1 year of age and adulthood (layer IV, *p* < 0.001; layer V, *p* = 0.030; layer VI, *p* = 0.007).

In area 36c, the distinct layers exhibited different volumetric developmental profiles (Figure 5E; layer × age group: *F*_(5.702,22.808)_ = 2.111, *p* = 0.094, η^2^_p_ = 0.345). Although statistical analyses did not reveal overall differences between age groups in the volume of layer I (*F*_(3,12)_ = 2.158, *p* = 0.146, η^2^_p_ = 0.350) or layer III (*F*_(3,12)_ = 2.155, *p* = 0.147, η^2^_p_ = 0.350), these two layers appeared to exhibit an early maturational profile with a major increase in volume between birth and 6 months of age (layer I, *p* = 0.052; layer III; *p* = 0.043), when they were 100% and 102% of their adult volume, respectively. Layer II (*F*_(3,12)_ = 5.818, *p* = 0.011, η^2^_p_ = 0.593), layer IV (*F*_(3,12)_ = 4.802, *p* = 0.020, η^2^_p_ = 0.546), layer V (*F*_(3,12)_ = 3.453, *p* = 0.051, η^2^_p_ = 0.463), and layer VI (*F*_(3,12)_ = 3.106, *p* = 0.067, η^2^_p_ = 0.437) exhibited a late maturation with a substantial increase in volume between 1 year of age and adulthood (layer II, *p* = 0.014; layer IV, *p* = 0.007; layer V, *p* = 0.038; layer VI, *p* = 0.047).

#### 3.1.2 Parahippocampal Cortex

##### 3.1.2.1 Subdivisions

Although the general statistical analysis suggested subtle differences in the volumetric development of areas TH and TF (Figure 5B; subdivision × age group: *F*_(3,12)_ = 3.677, *p* = 0.044, η^2^_p_ = 0.479), they both exhibited an early maturational profile. The volumes of area TF (*F*_(3,12)_ = 5.658, *p* = 0.012, η^2^_p_ = 0.586) and area TH (*F*_(3,12)_ = 6.292, *p* = 0.008, η^2^_p_ = 0.611) differed between age groups. Both areas exhibited a major increase in volume between birth and 6 months of age (TF, *p* = 0.006; TH, *p* = 0.018), when they were 102% and 95% of their adult volume, respectively.

##### 3.1.2.2 Layers Within Each Subdivision

In area TF, the distinct layers exhibited different volumetric developmental profiles (Figure 5F, layer × age group: *F*_(5.274,22.894)_ = 4.230, *p* = 0.006, η^2^_p_ = 0.514). Layer I (*F*_(3,12)_ = 8.881, *p* = 0.002, η^2^_p_ = 0.689), layer II (*F*_(3,12)_ = 12.357, *p* < 0.001, η^2^_p_ = 0.755), and layer VI (*F*_(3,12)_ = 4.678, *p* = 0.022, η^2^_p_ = 0.539) exhibited an early maturation, with a major volumetric increase between birth and 6 months of age (layer I, *p* = 0.001; layer II, *p* = 0.003; layer VI, *p* = 0.026), when they were 109%, 87%, and 92% of their adult volume, respectively. Although the global statistical analysis did not reveal age group differences in the volume of layer V (*F*_(3,12)_ = 2.462, *p* = 0.113, η^2^_p_ = 0.381), it also exhibited an early maturational profile, with an increase between birth and 6 months of age (*p* = 0.051), when it was 97% of the adult volume. Intriguingly, layer III exhibited a transient increase in volume (*F*_(3,12)_ = 5.098, *p* = 0.017, η^2^_p_ = 0.560), with an initial increase between birth and 6 months of age (*p* = 0.009), when it was 118% of its adult volume, followed by a decrease between 1 year of age and adulthood (*p* = 0.056). Finally, layer IV also exhibited some differences in volume between age groups (*F*_(3,12)_ = 3.549, *p* = 0.048, η^2^_p_ = 0.470), which reflected a gradual increase from birth to adulthood (*p* = 0.008).

In area TH, the distinct layers exhibited different volumetric developmental profiles (Figure 5G; layer × age group: *F*_(6.770,27.079)_ = 3.747, *p* = 0.006, η^2^_p_ = 0.484). Layer I (*F*_(3,12)_ = 6.130, *p* = 0.009, η^2^_p_ = 0.605), layer II (*F*_(3,12)_ = 10.534, *p* = 0.001, η^2^_p_ = 0.725), and layer III (*F*_(3,12)_ = 6.113, *p* = 0.009, η^2^_p_ = 0.604) exhibited an early maturation, with a major volumetric increase between birth and 6 months of age (layer I, *p* = 0.015; layer II, *p* = 0.004; layer III, *p* = 0.012), when they were 97%, 91%, and 100% of their adult volume, respectively. In contrast, statistical analyses did not reveal overall age group differences in the volume of layer V (*F*_(3,12)_ = 0.888, *p* = 0.475, η^2^_p_ = 0.182), which was 87% of the adult volume at birth, thus suggesting a very early maturational profile. Layer VI exhibited age group differences (*F*_(3,12)_ = 4.246, *p* = 0.029, η^2^_p_ = 0.515), which reflected a gradual increase from birth to adulthood (*p* = 0.005).

### 3.2 Neuron Number

**FIGURE 6:**
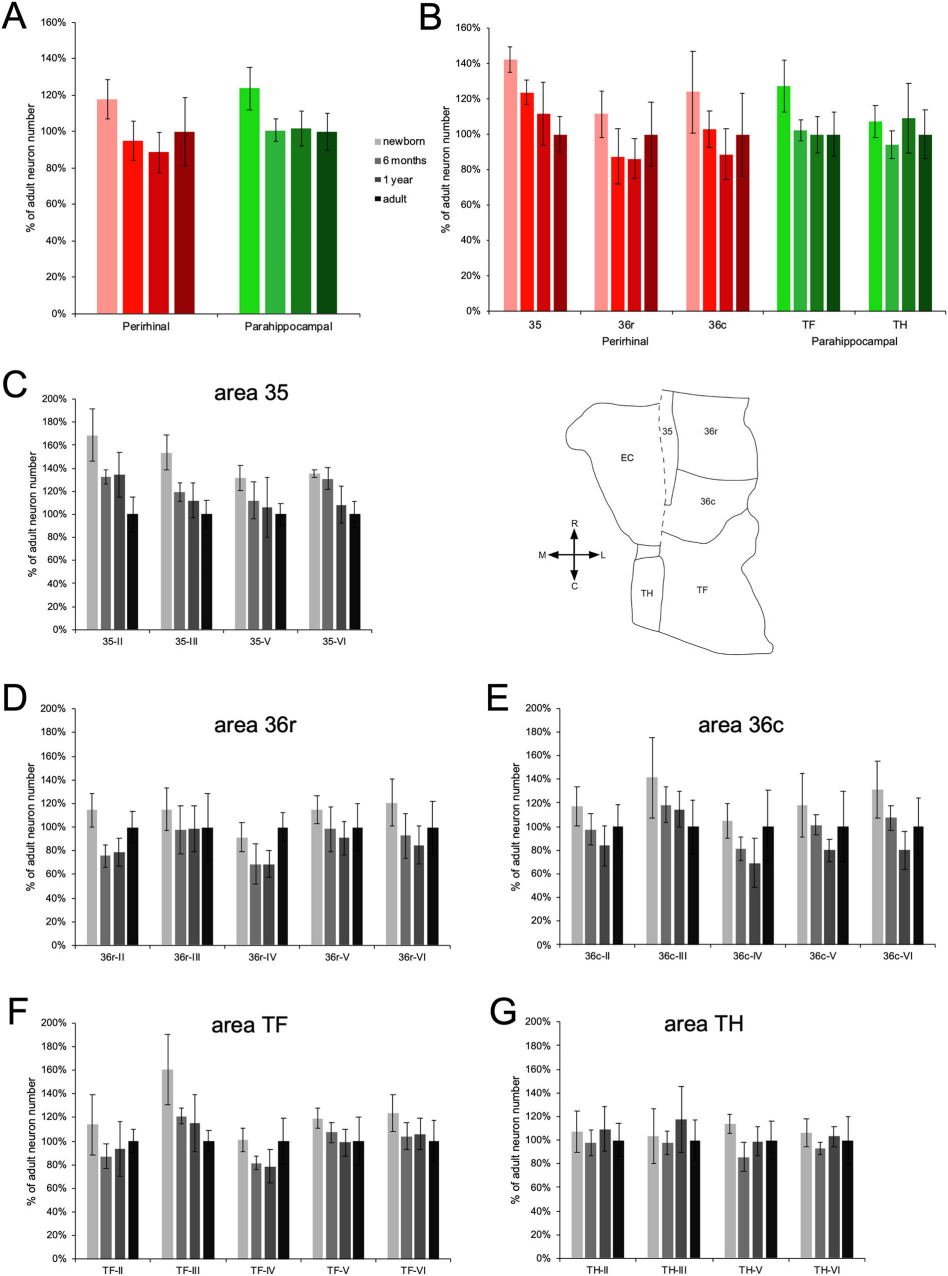
Percentage of adult neuron numbers in the distinct layers and subdivisions of the monkey perirhinal and parahippocampal cortices, at different postnatal ages. (A) Neuron numbers in the perirhinal cortex and parahippocampal cortex. (B) Neuron numbers in individual subdivisions. Neuron numbers in individual layers within each subdivision: (C) area 35; (D) area 36r; (E) area 36c; (F) area TF; (G) area TH. The unfolded map illustrates the relative position of each subdivision.

The numbers of neurons in the distinct layers and subdivisions of the perirhinal and parahippocampal cortices at different postnatal ages are presented in Table 4 and Figure 6. Both the perirhinal cortex (*F*_(3,12)_ = 3.475, *p* = 0.051, η^2^_p_ = 0.465) and the parahippocampal cortex (*F*_(3,12)_ = 5.635, *p* = 0.012, η^2^_p_ = 0.585) exhibited differences in neuron numbers between postnatal ages (Figure 6A). There were 24% more neurons in the perirhinal cortex at birth than at 6 months of age (*p* = 0.034) and 23% more neurons in the parahippocampal cortex at birth than at 6 months of age (*p* = 0.006). Neuron numbers remained stable from 6 months of age to adulthood in both cortices. Interestingly, the subdivisions of the perirhinal and parahippocampal cortices exhibited distinct age‐related differences in neuron numbers (Figure 6B, subdivision × age group: *F*_(7.259,29.035)_ = 3.040, *p* = 0.015, η^2^_p_ = 0.432), which are described below.

**TABLE 4:** Average number of neurons (± *SD*) in the different layers and subdivisions of the rhesus monkey perirhinal and parahippocampal cortices at four postnatal ages.

Area	Layer	Newborn	6 months	1 year	5–9 years
35	II	69,115 ± 9,324	54,180 ± 2,468	54,968 ± 7,814	40,922 ± 6,366
	III	120,872 ± 11,967	93,951 ± 6,552	88,288 ± 11,719	78,648 ± 9,447
	V	111,384 ± 9,369	94,964 ± 13,584	89,961 ± 22,068	84,795 ± 7,801
	VI	193,042 ± 5,274	186,702 ± 13,455	154,560 ± 22,628	142,569 ± 16,447
	All layers	494,413 ± 25,170	429,797 ± 23,417	387,777 ± 62,207	346,934 ± 34,303
36r	II	886,662 ± 109,866	586,713 ± 70,986	609,292 ± 91,429	775,309 ± 99,867
	III	1,222,653 ± 190,908	1,041,057 ± 220,457	1,054,485 ± 205,420	1,065,249 ± 300,898
	IV	523,527 ± 70,496	393,301 ± 98,891	392,413 ± 64,029	571,951 ± 69,981
	V	784,883 ± 79,657	672,821 ± 127,866	620,901 ± 98,424	684,818 ± 134,940
	VI	461,408 ± 75,955	353,715 ± 71,110	324,385 ± 61,903	382,282 ± 84,649
	All layers	3,879,133 ± 453,580	3,047,607 ± 540,183	3,001,476 ± 390,276	3,479,609 ± 628,045
36c	II	525,265 ± 75,198	437,516 ± 60,483	375,995 ± 75,527	448,180 ± 81,400
	III	809,611 ± 195,282	677,144 ± 85,073	655,283 ± 85,954	571,904 ± 129,130
	IV	338,937 ± 47,864	262,693 ± 32,390	222,867 ± 66,678	322,541 ± 98,295
	V	527,091 ± 119,209	452,736 ± 36,120	356,626 ± 40,802	446,022 ± 132,723
	VI	328,567 ± 59,995	268,412 ± 25,597	200,264 ± 39,871	249,957 ± 60,458
	All layers	2,529,471 ± 469,023	2,098,501 ± 208,709	1,811,035 ± 293,989	2,038,604 ± 476,416
Perirhinal cortex		6,903,017 ± 645,281	5,575,905 ± 632,022	5,200,288 ± 640,862	5,865,147 ± 1,109,303
TF	II	1,452,771 ± 324,607	1,108,119 ± 133,471	1,192,669 ± 296,829	1,274,438 ± 125,680
	III	2,686,767 ± 497,325	2,026,128 ± 110,914	1,931,066 ± 403,226	1,676,232 ± 156,773
	IV	951,191 ± 92,346	768,642 ± 55,129	741,421 ± 135,972	942,216 ± 185,835
	V	1,651,291 ± 123,037	1,484,222 ± 120,769	1,367,847 ± 158,817	1,383,945 ± 287,091
	VI	729,046 ± 92,133	612,049 ± 67,284	624,065 ± 76,169	588,363 ± 104,078
	All layers	7,471,066 ± 870,021	5,999,160 ± 341,888	5,857,068 ± 617,227	5,865,194 ± 733,304
TH	II	286,374 ± 46,159	260,876 ± 29,027	292,769 ± 50,696	267,617 ± 38,507
	III	585,608 ± 129,805	554,847 ± 54,346	665,854 ± 158,621	567,100 ± 97,598
	V	401,413 ± 28,726	301,938 ± 43,492	348,894 ± 43,873	352,391 ± 56,769
	VI	142,042 ± 16,092	124,805 ± 7,172	137,700 ± 11,337	133,689 ± 26,978
	All layers	1,415,437 ± 119,139	1,242,466 ± 103,376	1,445,217 ± 260,511	1,320,797 ± 181,500
Parahippocampal cortex		8,886,503 ± 853,411	7,241,626 ± 433,813	7,302,285 ± 685,231	7,185,991 ± 732,814

#### 3.2.1 Perirhinal Cortex

##### 3.2.1.1 Subdivisions

Statistical analyses revealed age group differences in the number of neurons in area 35 (*F*_(3,12)_ = 10.189, *p* = 0.001, η^2^_p_ = 0.718), but not in area 36r (*F*_(3,12)_ = 2.611, *p* = 0.100, η^2^_p_ = 0.395) or area 36c (*F*_(3,12)_ = 2.498, *p* = 0.109, η^2^_p_ = 0.384). In area 35, there were 43% more neurons at birth than in adulthood (*p* < 0.001). The decrease in neuron numbers occurred gradually but was most prominent between birth and 6 months of age (*p* = 0.039).

##### 3.2.1.2 Layers Within Each Subdivision

In area 35, the distinct layers exhibited different developmental changes in neuron number (Figure 6C; layer × age group: *F*_(9,36)_ = 3.721, *p* = 0.002, η^2^_p_ = 0.482). Layer II (*F*_(3,12)_ = 10.906, *p* < 0.001, η^2^_p_ = 0.732) and layer III (*F*_(3,12)_ = 12.692, *p* < 0.001, η^2^_p_ = 0.760) had 69% and 54% more neurons at birth than in adulthood, respectively (layer II, *p* < 0.001; layer III, *p* < 0.001). Layer VI (*F*_(3,12)_ = 9.674, *p* = 0.002, η^2^_p_ = 0.707) had 35% more neurons at birth than in adulthood (*p* < 0.001), but in contrast to the superficial layers this decrease in neuron number occurred mainly between 6 months and 1 year of age (*p* = 0.014). Although statistical analyses did not reveal overall age‐related differences in neuron number in layer V (*F*_(3,12)_ = 2.586, *p* = 0.102, η^2^_p_ = 0.393), there were 31% more neurons at birth than in adulthood (*p* = 0.022).

In area 36r, the distinct layers did not exhibit statistically significant differences in neuron number between age groups (Figure 6D, age group: *F*_(3,12)_ = 2.603, *p* = 0.100, η^2^_p_ = 0.394; layer × age group: *F*_(5.527,22.108)_ = 1.146, *p* = 0.368, η^2^_p_ = 0.223), and the number of neurons did not differ between birth and adulthood for any of the layers.

In area 36c, although the general analysis suggested different age‐related differences in neuron number in distinct layers (Figure 6E; layer × age group: *F*_(12,48)_ = 2.566, *p* = 0.010, η^2^_p_ = 0.391), unlike area 35 and similar to area 36r, most layers of area 36c did not exhibit a clear pattern of age‐related differences in neuron number (layer II: *F*_(3,12)_ = 2.774, *p* = 0.087, η^2^_p_ = 0.410; layer III: *F*_(3,12)_ = 2.235, *p* = 0.137, η^2^_p_ = 0.358; layer IV: *F*_(3,12)_ = 2.648, *p* = 0.097, η^2^_p_ = 0.398; layer V: *F*_(3,12)_ = 2.239, *p* = 0.136, η^2^_p_ = 0.359). Only layer VI exhibited an overall difference in neuron number between age groups (*F*_(3,12)_ = 4.732, *p* = 0.021, η^2^_p_ = 0.542), which reflected a 31% decrease in neuron number between birth and adulthood (*p* = 0.042). In addition, although the global analysis did not reach statistical significance, it is interesting to note that layer III of area 36c exhibited a gradual decrease (42%) of neuron number between birth and adulthood (*p* = 0.025), which resembled that observed in layer III of areas 35 and TF (see below).

#### 3.2.2 Parahippocampal Cortex

##### 3.2.2.1 Subdivisions

The number of neurons differed between age groups in area TF (*F*_(3,12)_ = 5.496, *p* = 0.013, η^2^_p_ = 0.579), but not in area TH (*F*_(3,12)_ = 1.087, *p* = 0.392, η^2^_p_ = 0.214; subdivision × age group: *F*_(3,12)_ = 4.777, *p* = 0.020, η^2^_p_ = 0.544). In area TF, there were 25% more neurons at birth than at 6 months of age (*p* = 0.009) when neuron number was similar to that observed in adulthood.

##### 3.2.2.2 Layers Within Each Subdivision

In area TF, the distinct layers exhibited different developmental profiles in neuron number (Figure 6F; layer × age group: *F*_(7.238,28.952)_ = 3.427, *p* = 0.008, η^2^_p_ = 0.461). Layer III exhibited differences between age groups (*F*_(3,12)_ = 6.640, *p* = 0.007, η^2^_p_ = 0.624), whereas the other layers did not (layer II: *F*_(3,12)_ = 1.525, *p* = 0.258, η^2^_p_ = 0.276; layer IV: *F*_(3,12)_ = 3.067, *p* = 0.069, η^2^_p_ = 0.434; layer V: *F*_(3,12)_ = 1.976, *p* = 0.171, η^2^_p_ = 0.331; layer VI: *F*_(3,12)_ = 2.090, *p* = 0.155, η^2^_p_ = 0.343). In layer III, there were 60% more neurons at birth than in adulthood (*p* = 0.001). The decrease in neuron number occurred gradually but was most prominent between birth and 6 months of age (*p* = 0.016).

In area TH, the distinct layers did not exhibit age‐related differences in neuron number (Figure 6G; age groups: *F*_(3,12)_ = 1.087, *p* = 0.392, η^2^_p_ = 0.214; layer × age group: *F*_(4.222,16.890)_ = 0.938, *p* = 0.470, η^2^_p_ = 0.190).

### 3.3 Neuronal Soma Size

**FIGURE 7:**
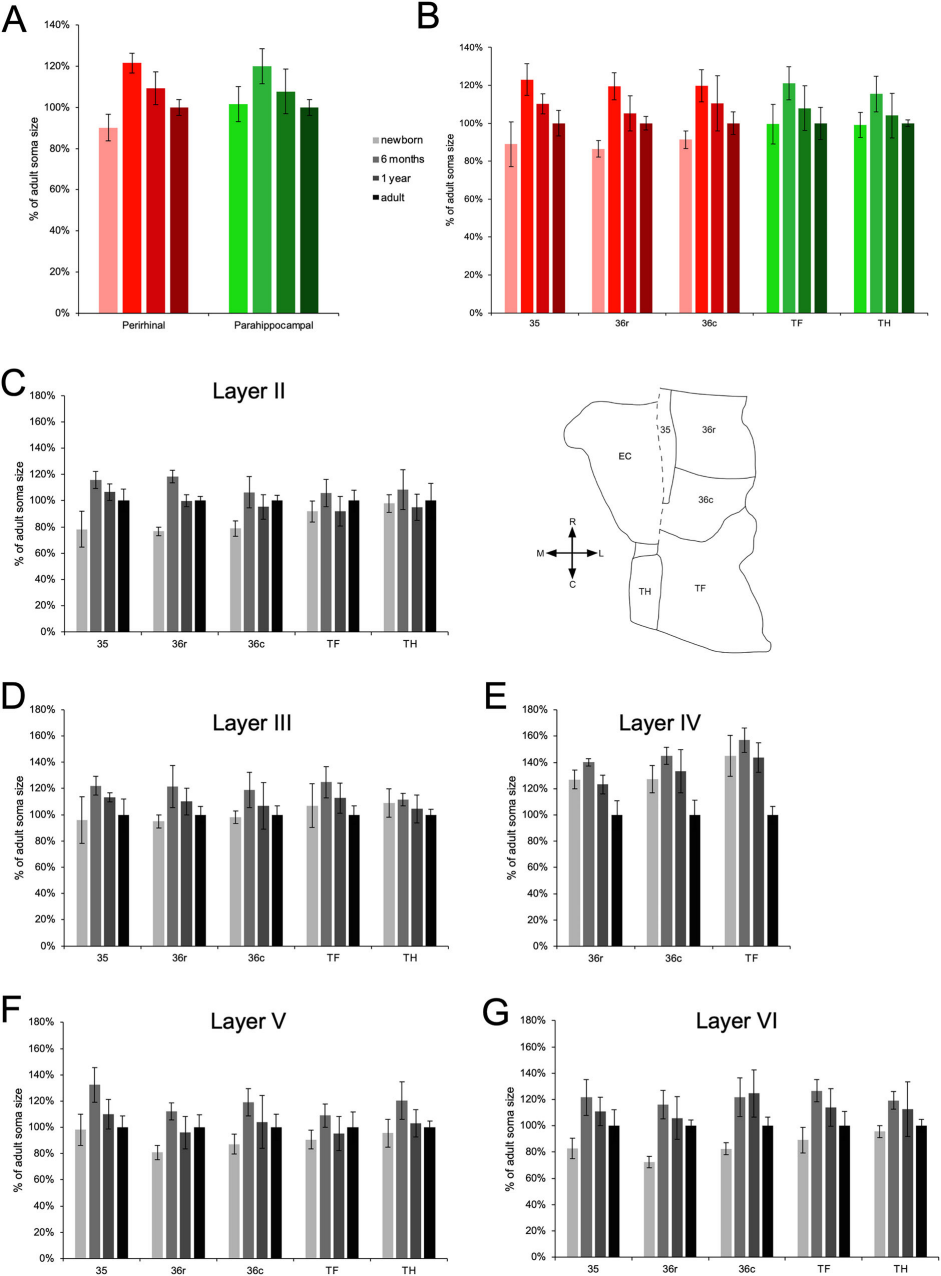
Percentage of adult neuronal soma size in the distinct layers and subdivisions of the monkey perirhinal and parahippocampal cortices, at different postnatal ages. (A) Neuronal soma size in the perirhinal cortex and parahippocampal cortex. (B) Neuronal soma size in individual subdivisions. Neuronal soma sizes in individual layers across all subdivisions: (C) layer II; (D) layer III; (E) layer IV; (F) layer V; (G) layer VI. The unfolded map illustrates the relative position of each subdivision.

The average neuronal soma size in the distinct layers and subdivisions of the perirhinal and parahippocampal cortices at different postnatal ages are presented in Tables 5 and 6 and Figure 7.

**TABLE 5:** Average neuronal soma size (± *SD*) in the different layers and subdivisions of the rhesus monkey perirhinal and parahippocampal cortices at four postnatal ages.

Area	Layer	Newborn	6 months	1 year	5–9 years
35	II	1,538 ± 270	2,276 ± 126	2,094 ± 126	1,966 ± 170
	III	1,767 ± 328	2,251 ± 132	2,088 ± 61	1,844 ± 223
	V	1,951 ± 237	2,630 ± 266	2,190 ± 223	1,988 ± 172
	VI	1,323 ± 126	1,941 ± 219	1,773 ± 172	1,597 ± 196
	All layers	1,645 ± 216	2,275 ± 155	2,036 ± 97	1,849 ± 124
36r	II	1,076 ± 46	1,659 ± 66	1,402 ± 64	1,404 ± 44
	III	1,469 ± 76	1,878 ± 245	1,701 ± 159	1,546 ± 96
	IV	986 ± 55	1,089 ± 22	957 ± 56	777 ± 85
	V	1,337 ± 89	1,853 ± 108	1,587 ± 207	1,654 ± 156
	VI	1,120 ± 68	1,797 ± 166	1,638 ± 252	1,549 ± 71
	All layers	1,198 ± 61	1,655 ± 99	1,457 ± 128	1,386 ± 50
36c	II	1,085 ± 82	1,466 ± 164	1,312 ± 126	1,378 ± 58
	III	1,505 ± 72	1,822 ± 207	1,638 ± 270	1,534 ± 107
	IV	911 ± 73	1,038 ± 47	955 ± 119	716 ± 82
	V	1,365 ± 119	1,861 ± 162	1,628 ± 318	1,564 ± 158
	VI	1,172 ± 64	1,729 ± 207	1,769 ± 257	1,420 ± 92
	All layers	1,208 ± 62	1,583 ± 112	1,460 ±193	1,322 ± 80
Perirhinal cortex		1,329 ± 97	1,806 ± 73	1,624 ± 120	1,495 ± 58
TF	II	995 ± 86	1,148 ± 113	998 ± 122	1,083 ± 87
	III	1,319 ± 204	1,539 ± 148	1,389 ± 141	1,233 ± 83
	IV	849 ± 92	919 ± 55	842 ± 67	586 ± 37
	V	1,292 ± 101	1,554 ± 126	1,357 ± 186	1,425 ± 164
	VI	1,264 ± 137	1,796 ± 121	1,615 ± 200	1,418 ± 153
	All layers	1,144 ± 121	1,391 ± 99	1,240 ± 134	1,149 ± 97
TH	II	1,237 ± 84	1,369 ± 190	1,197 ± 126	1,263 ± 166
	III	1,506 ± 148	1,541 ± 67	1,446 ± 146	1,383 ± 61
	V	1,661 ± 184	2,090 ± 248	1,790 ± 182	1,738 ± 81
	VI	1,428 ± 65	1,783 ± 99	1,684 ± 310	1,494 ± 74
	All layers	1,458 ± 97	1,696 ± 139	1,529 ± 173	1,470 ± 25
Parahippocampal cortex		1,283 ± 105	1,527 ± 113	1,369 ± 143	1,291 ± 48

**TABLE 6:** Results of the statistical analyses on neuronal soma size and attribution of distinct maturation profiles to the different layers and subdivisions of the monkey perirhinal and parahippocampal cortices.

Area	Layer	Profile^a^	*F*_(2,13)_	*p*	η^2^_p_	Post hoc comparisons with *p* < 0.05
35	II	Transient‐increase	11.790	< 0.001	0.747	Newborn < other ages; 6 months > 5–9 years
	III	Transient	4.444	0.026	0.526	Newborn < 6 months > 5–9 years
	V	Transient	7.555	0.004	0.654	6 months > other ages
	VI	Transient‐increase	8.494	0.003	0.680	Newborn < 6 months ‐ 1 year; 6 months > 5–9 years
	All layers	Transient‐increase	12.074	< 0.001	0.751	Newborn < 6 months > 1 year ‐ 5–9 years
36r	II	Transient‐increase	73.188	< 0.001	0.948	Newborn < other ages; 6 months > 1 year ‐ 5–9 years
	III	Transient	5.211	0.016	0.566	Newborn < 6 months > 5–9 years
	IV	Transient‐decrease	19.511	< 0.001	0.830	6 months > newborn ‐ 1 year; 5–9 years < other ages
	V	Transient‐increase	8.347	0.003	0.676	Newborn < other ages; 6 months > 1 year
	VI	Transient‐increase	13.323	< 0.001	0.769	Newborn < other ages; 6 months > 5–9 years
	All layers	Transient‐increase	17.635	< 0.001	0.815	Newborn < other ages; 6 months > 1 year ‐ 5–9 years
36c	II	Early increase	7.987	0.003	0.666	Newborn < 6 months ‐ 1 year ‐ 5–9 years
	III	Transient	2.490	0.110	0.384	Newborn < 6 months > 5–9 years
	IV	Transient‐decrease	10.536	0.001	0.725	Newborn ‐ 6 months ‐ 1 year > 5–9 years
	V	Transient	4.030	0.034	0.502	Newborn < 6 months
	VI	Transient‐increase	10.385	0.001	0.722	Newborn < 6 months ‐ 1 year > 5–9 years
	All layers	Transient	7.115	0.005	0.640	Newborn < 6 months > 5–9 years
Perirhinal cortex		Transient‐increase	19.855	< 0.001	0.832	Newborn < 6 months > 1 year > 5–9 years
TF	II	Very early	2.061	0.159	0.340	—
	III	Transient	2.971	0.074	0.426	6 months > 5–9 years
	IV	Late decrease	19.875	< 0.001	0.832	Newborn ‐ 6 months ‐ 1 year > 5–9 years
	V	Very early	2.291	0.130	0.364	—
	VI	Transient	8.865	0.002	0.689	Newborn < 6 months ‐ 1 year; 6 months > 5–9 years
	All layers	Transient	4.125	0.032	0.508	Newborn < 6 months > 5–9 years
TH	II	Very early	0.998	0.427	0.200	—
	III	Very early	1.502	0.264	0.273	—
	V	Transient	4.175	0.031	0.511	6 months > other ages
	VI	Transient	3.737	0.042	0.483	Newborn < 6 months > 5–9 years
	All layers	Transient	3.251	0.060	0.448	Newborn < 6 months > 5–9 years
Parahippocampal cortex		Transient	4.746	0.021	0.543	Newborn < 6 months > 5–9 years

^a^ Profiles were defined semiquantitatively, based on both statistical tests and general trends of age group differences.

The average neuronal soma size in the perirhinal cortex (*F*_(3,12)_ = 19.855, *p* < 0.001, η^2^_p_ = 0.832) and parahippocampal cortex (*F*_(3,12)_ = 4.746, *p* = 0.021, η^2^_p_ = 0.543) differed between age groups. The developmental changes in neuronal soma size differed between the perirhinal and parahippocampal cortices (cortex × age group: *F*_(3,12)_ = 7.819, *p* = 0.004, η^2^_p_ = 0.662). Neuronal soma size exhibited an increase between birth and 6 months of age in the perirhinal cortex (*p* < 0.001), followed by a gradual decrease between 6 months and adulthood (*p* < 0.001) and a larger average neuronal soma size in adulthood than at birth (*p* = 0.039). Similarly, neuronal soma size exhibited an increase between birth and 6 months of age in the parahippocampal cortex (*p* = 0.009), followed by a gradual decrease between 6 months and adulthood (*p* = 0.005). However, in contrast to the perirhinal cortex, average neuronal soma size did not differ between birth and adulthood in the parahippocampal cortex (*p* = 0.795). Following the developmental profile descriptions of Piguet et al. (2020), average neuronal soma size exhibited a transient ‐ increase profile in the perirhinal cortex, whereas it exhibited a transient profile in the parahippocampal cortex.

However, the subdivisions of the perirhinal and parahippocampal cortices exhibited different postnatal changes in neuronal soma size (Figure 7B; subdivision × age group: *F*_(12,48)_ = 2.583, *p* = 0.010, η^2^_p_ = 0.392). Interestingly, we observed a rostrocaudal gradient of age‐related differences in neuronal soma size across subdivisions, with caudal subdivisions exhibiting more subtle differences compared to rostral subdivisions. Accordingly, postnatal changes in neuronal soma size did not differ between areas 35, 36r, and 36c of the perirhinal cortex (subdivision × age group: *F*_(6,24)_ = 1.263, *p* = 0.311, η^2^_p_ = 0.240), or between areas TF and TH of the parahippocampal cortex (subdivision × age group, *F*_(3,12)_ = 0.088, *p* = 0.965, η^2^_p_ = 0.021). However, age‐related differences in neuronal soma size varied between the distinct layers of the perirhinal cortex (layer × age group: *F*_(6.678,26.712)_ = 7.497, *p* < 0.001, η^2^_p_ = 0.652) and between the distinct layers of the parahippocampal cortex (layer × age group: *F*_(12,48)_ = 5.727, *p* < 0.001, η^2^_p_ = 0.589). Age‐related differences were thus further analyzed per layer and compared between individual subdivisions (Figure 7C–G).

#### 3.3.1 Perirhinal Cortex

##### 3.3.1.1 Layer II

Neuronal soma size differed between age groups in layer II of area 35 (*F*_(3,12)_ = 11.790, *p* < 0.001, η^2^_p_ = 0.747), area 36r (*F*_(3,12)_ = 73.188, *p* < 0.001, η^2^_p_ = 0.948), and area 36c (*F*_(3,12)_ = 7.987, *p* = 0.003, η^2^_p_ = 0.666). Although age‐related differences in neuronal soma size did not differ between areas 35, 36r, and 36c (subdivision × age group: *F*_(6,24)_ = 1.735, *p* = 0.156, η^2^_p_ = 0.303), there were subtle differences. In all three subdivisions, neuronal soma size exhibited an increase between birth and 6 months of age (area 35, *p* < 0.001; area 36r, *p* < 0.001; area 36c, *p* < 0.001). In areas 35 and 36r, this initial increase was followed by a decrease, either between 6 months and 1 year for area 36r (*p* < 0.001) or more gradually between 6 months and adulthood for area 35 (*p* = 0.034). In contrast, there was no difference in neuronal soma size between 6 months of age and adulthood in area 36c (*p* = 0.301), which therefore better reflected an early increase and thus an intermediate profile between those observed in areas 35 and 36r and those observed in areas TF and TH (see below). Neuronal soma size increased between birth and adulthood in the three subdivisions (area 35, *p* = 0.006; area 36r, *p* = < 0.001; area 36c, *p* = 0.004).

##### 3.3.1.2 Layer III

Neuronal soma size differed between age groups in layer III of area 35 (*F*_(3,12)_ = 4.444, *p* = 0.026, η^2^_p_ = 0.526) and area 36r (*F*_(3,12)_ = 5.211, *p* = 0.016, η^2^_p_ = 0.566), but not in area 36c (*F*_(3,12)_ = 2.490, *p* = 0.110, η^2^_p_ = 0.384). Nevertheless, age‐related differences in neuronal soma size did not differ between areas 35, 36r, and 36c (subdivision × age group: *F*_(6,24)_ = 0.293, *p* = 0.934, η^2^_p_ = 0.068) and corresponded to a transient maturational profile. Neuronal soma size exhibited an increase between birth and 6 months of age in area 35 (*p* = 0.007), area 36r (*p* = 0.003), and area 36c (*p* = 0.030), followed by a decrease between 6 months and adulthood (area 35, *p* = 0.018; area 36r, *p* = 0.012; area 36c, *p* = 0.045), when it returned to a size similar to that observed at birth.

##### 3.3.1.3 Layer IV

Neuronal soma size differed between age groups in layer IV of area 36r (*F*_(3,12)_ = 19.511, *p* < 0.001, η^2^_p_ = 0.830) and area 36c (*F*_(3,12)_ = 10.536, *p* = 0.001, η^2^_p_ = 0.725), where this layer is present. Age‐related differences in neuronal soma size did not differ between areas 36r and 36c (subdivision × age group: *F*_(3,12)_ = 0.376, *p* = 0.772, η^2^_p_ = 0.086), and corresponded to a transient‐decrease maturation profile. Neuronal soma size exhibited an increase between birth and 6 months of age in area 36r (*p* = 0.029) and area 36c, (*p* = 0.054), followed by a decrease between 6 months and adulthood (area 36r, *p* < 0.001; area 36c (*p* < 0.001). Neuronal soma size was smaller in adulthood than at birth in both area 36r (*p* = < 0.001) and area 36c (*p* = 0.007).

##### 3.3.1.4 Layer V

Neuronal soma size differed between age groups in layer V of area 35 (*F*_(3,12)_ = 7.555, *p* = 0.004, η^2^_p_ = 0.654), area 36r (*F*_(3,12)_ = 8.347, *p* = 0.003, η^2^_p_ = 0.676), and area 36c (*F*_(3,12)_ = 4.030, *p* = 0.034, η^2^_p_ = 0.502). Age‐related differences in neuronal soma size did not differ between areas 35, 36r, and 36c (subdivision × age group: *F*_(6,24)_ = 1.465, *p* = 0.232, η^2^_p_ = 0.268). However, neuronal soma size exhibited a transient maturational profile in layer V of areas 35 and 36c, and a transient‐increase profile in area 36r. Neuronal soma size exhibited an increase between birth and 6 months of age in area 35 (*p* = 0.001), area 36r (*p* < 0.001), and area 36c (*p* = 0.005), which was followed by a decrease between 6 months and 1 year of age in area 35 (*p* = 0.018) and area 36r (36r, *p* = 0.025), and between 6 months of age and adulthood in area 36c (*p* = 0.062). Neuronal soma size was larger in adulthood than at birth in area 36r (*p* = 0.010), but not in area 35 (*p* = 0.820) or area 36c (*p* = 0.192).

##### 3.3.1.5 Layer VI

Neuronal soma size differed between age groups in layer VI of area 35 (*F*_(3,12)_ = 8.494, *p* = 0.003), area 36r (*F*_(3,12)_ = 13.323, *p* < 0.001, η^2^_p_ = 0.769), and area 36c (*F*_(3,12)_ = 10.385, *p* = 0.001, η^2^_p_ = 0.722). Age‐related differences in neuronal soma size did not differ between areas 35, 36r, and 36c (subdivision × age group: *F*_(6,24)_ = 1.264, *p* = 0.311, η^2^_p_ = 0.24) and corresponded to a transient‐increase maturational profile. Neuronal soma size exhibited an increase between birth and 6 months of age in area 35 (*p* < 0.001), area 36r (*p* < 0.001), and area 36c (*p* < 0.001), which was followed by a decrease between 6 months of age and adulthood in area 35 (*p* = 0.020) and area 36r (*p* = 0.047), and between 1 year of age and adulthood in area 36c (*p* = 0.015). Neuronal soma size was larger in adulthood than at birth in area 35 (*p* = 0.054), area 36r (*p* = 0.002), and area 36c (*p* = 0.067).

#### 3.3.2 Parahippocampal Cortex

##### 3.3.2.1 Layer II

Neuronal soma size did not differ between age groups in layer II of area TF (*F*_(3,12)_ = 2.061, *p* = 0.159, η^2^_p_ = 0.340) or area TH (*F*_(3,12)_ = 0.998, *p* = 0.427, η^2^_p_ = 0.200). The developmental profile of neuronal soma size was thus similar for the two areas (subdivision × age group: *F*_(3,12)_ = 0.170, *p* = 0.915, η^2^_p_ = 0.041) and reflected an early maturational profile. Nevertheless, layer II of area TF exhibited a maturational profile in between that of area TH and that of area 36c, with an increase in neuronal soma size between birth and 6 months of age (*p* = 0.057), followed by a decrease between 6 months and 1 year of age (*p* = 0.061). However, in contrast to area 36c, neuronal soma size did not differ between birth and adulthood in layer II of area TF (*p* = 0.248).

##### 3.3.2.2 Layer III

Neuronal soma size did not differ between age groups in layer III of area TF (*F*_(3,12)_ = 2.971, *p* = 0.074, η^2^_p_ = 0.426) or area TH (*F*_(3,12)_ = 1.502, *p* = 0.264, η^2^_p_ = 0.273). The developmental profile of neuronal soma size was thus similar for the two areas (subdivision × age group: *F*_(3,12)_ = 2.504, *p* = 0.109, η^2^_p_ = 0.385) and reflected an early maturational profile. Nevertheless, similar to area 36c, layer III of area TF exhibited a transient maturational profile, with an initial increase in neuronal soma size between birth and 6 months of age (*p* = 0.061), followed by a gradual decrease from 6 months of age to adulthood (*p* = 0.014). However, in contrast to area 36c, neuronal soma size did not differ between birth and adulthood in layer II of area TF (*p* = 0.434).

##### 3.3.2.3 Layer IV

Neuronal soma size differed between age groups in layer IV of area TF (*F*_(3,12)_ = 19.875, *p* < 0.001, η^2^_p_ = 0.832), with a marked decrease between 1 year of age and adulthood (*p* < 0.001), thus reflecting a late decrease maturation profile.

##### 3.3.2.4 Layer V

Neuronal soma size did not differ between age groups in layer V of area TF (*F*_(3,12)_ = 2.291, *p* = 0.130, η^2^_p_ = 0.364), but it differed in area TH (*F*_(3,12)_ = 4.175, *p* = 0.031, η^2^_p_ = 0.511). Although the developmental profiles of neuronal soma size were not clearly distinct in areas TF and TH (subdivision × age group: *F*_(3,12)_ = 1.816, *p* = 0.198, η^2^_p_ = 0.312), layer V exhibited a very early maturational profile in area TF and a transient maturational profile in area TH. Neuronal soma size exhibited an increase between birth and 6 months of age in both area TF (*p* = 0.028) and area TH (*p* = 0.006) followed by a decrease between 6 months and 1 year of age in area TF (*p* = 0.084) and area TH *(p* = 0.040), when it returned to a size similar to that observed at birth in area TF (*p* = 0.230) and area TH (*p* = 0.564).

##### 3.3.2.5 Layer VI

Neuronal soma size differed between age groups in layer VI of area TF (*F*_(3,12)_ = 8.865, *p* = 0.002, η^2^_p_ = 0.689) and area TH (*F*_(3,12)_ = 3.737, *p* = 0.042, η^2^_p_ = 0.483). Age‐related differences in neuronal soma size were similar in areas TF and TH (subdivision × age group: *F*_(3,12)_ = 1.251, *p* = 0.335, η^2^_p_ = 0.238) and corresponded to a transient maturational profile. Neuronal soma size exhibited an increase between birth and 6 months of age in area TF (*p* < 0.001) and area TH (*p* = 0.012), followed by a decrease between 6 months of age and adulthood in area TF (*p* = 0.005) and area TH (*p* = 0.034), when it returned to a size similar to that observed at birth in area TF (*p* = 0.188) and area TH (*p* = 0.591).

### 3.4 Immature and Mature Neurons in Layer II of Area 36

**FIGURE 8:**
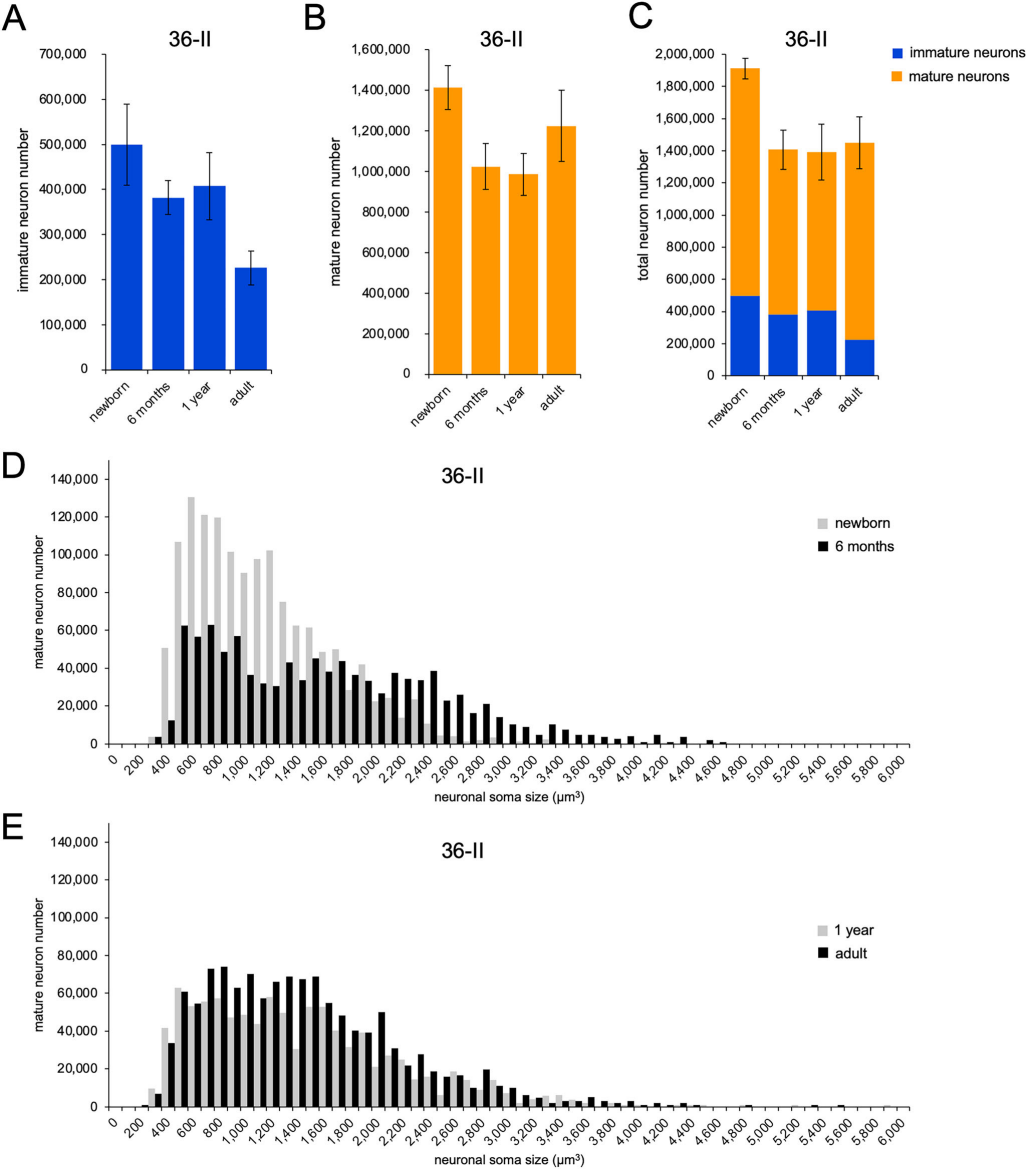
Number of immature and mature neurons in layer II of area 36 (including areas 36r and 36c) of the perirhinal cortex, at different postnatal ages. (A) Number of immature neurons. (B) Number of mature neurons. (C) Total number of neurons. (D) Neuronal soma size distribution of mature neurons at birth (gray) and 6 months (black). (E) Neuronal soma size distribution of mature neurons at 1 year (gray) and in adulthood (black).

Layer II of the perirhinal cortex contains a population of immature neurons, whose number can increase following developmental perturbations, such as neonatal hippocampal damage (Villard et al. 2023). We therefore also estimated the number of immature neurons in layer II of area 36 (including areas 36r and 36c). The number of immature neurons differed between age groups (Figure 8A; *F*_(3,12)_ = 12.592, *p* < 0.001, η^2^_p_ = 0.759), and exhibited a two‐step decrease, with 24% fewer immature neurons at 6 months of age than at birth (*p* = 0.023), and 45% fewer immature neurons in adulthood than at 1 year of age (*p* = 0.002). In parallel, the number of mature neurons in layer II of area 36 also differed between groups (Figure 8B; *F*_(3,12)_ = 9.386, *p* = 0.002, η^2^_p_ = 0.701). The number of mature neurons exhibited a decrease between birth and 6 months of age (*p* = 0.001), remained stable between 6 months and 1 year of age (*p* = 0.676), and exhibited an increase between 1 year of age and adulthood (*p* = 0.022). However, the difference in mature neuron number between birth and adulthood did not reach the predefined level of statistical significance (*p* = 0.060). Interestingly, the sum of the numbers of immature and mature neurons in layer II of area 36 exhibited age group differences (Figure 8C; *F*_(3,12)_ = 13.362, *p* < 0.001, η^2^_p_ = 0.770); there was a decrease between birth and 6 months of age (*p* < 0.001), followed by a stable number of neurons between 6 months and adulthood (*p* = 0.659). The distribution of soma size of mature neurons in layer II of area 36 (Figure 8D,E) reveals a decrease of the number of small mature neurons between birth and 6 months of age, whereas the increase of the number of mature neurons between 1 year and adulthood was not restricted to neurons of any specific soma size.

## 4 Discussion

The goal of this study was to characterize the postnatal structural development of the monkey perirhinal and parahippocampal cortices, by providing estimates of volume, neuron number, and neuronal soma size for each subdivision and layer at 1 day, 6 months, 1 year, and 5–9 years of age. Consistent with previous findings showing the differential maturation of distinct regions and layers of the hippocampal formation (Jabès et al. 2010, 2011), including the entorhinal cortex (Piguet et al. 2020), our current results showed that the parahippocampal cortex exhibits an overall earlier maturation than the perirhinal cortex. These volumetric changes are accompanied by a transient increase in neuronal soma size between birth and 6 months of age, followed by a relative decrease of soma size into adulthood in most layers. Moreover, we found a decrease in neuron numbers in all layers of area 35 and in layer III of area TF, especially between birth and 6 months of age. Together, these findings support the theory that distinct functional circuits of the medial temporal lobe mature at different times during development and may underlie the emergence of different “hippocampus‐dependent” memory processes during the first years of life (Lavenex et al. 2024; Lavenex and Banta Lavenex 2013).

### 4.1 Developmental Changes in Volume

The parahippocampal cortex exhibited an overall early volumetric maturation between birth and 6 months of age, whereas the perirhinal cortex exhibited a more protracted maturation continuing beyond 1 year of age. The differential maturation of these two cortices is consistent with the early maturation of the caudal portion of the entorhinal cortex and the late maturation of the rostral portion of the entorhinal cortex (Piguet et al. 2020), with which the parahippocampal cortex and the perirhinal cortex are highly interconnected, respectively (Suzuki and Amaral 1994b). Consistent with our findings in monkeys, the rat postrhinal projections toward the medial entorhinal cortex mature earlier that the projections toward the lateral entorhinal cortex (Lagartos‐Donate et al. 2022). Altogether, these findings support the view that the two main functional circuits within the medial temporal lobe memory system, which process different types of information, mature at different times during postnatal development: an early maturing circuit including the parahippocampal cortex and the caudal portion of the entorhinal cortex involved in spatial information processing, and a late‐maturing circuit including the perirhinal cortex and the rostral portion of the entorhinal cortex involved in object information processing.

#### 4.1.1 Subdivisions

The analysis of individual subdivisions within the perirhinal and parahippocampal cortices confirmed this rostrocaudal gradient of volumetric maturation. With the exception of area 35, which exhibited a unique profile of very early volumetric maturation, both areas TF and TH of the parahippocampal cortex exhibited an early maturation between birth and 6 months of age, while area 36r of the perirhinal cortex exhibited a late maturation after 1 year of age. Interestingly, area 36c of the perirhinal cortex exhibited a developmental profile in between the late development of area 36r and the early development of areas TF and TH. This rostrocaudal gradient of maturation is consistent with the maturational profiles previously reported in the subdivisions of the entorhinal cortex (Piguet et al. 2020): the caudal areas Elc, Ec, and Ecl exhibit an early development between birth and 6 months of age, whereas the rostral areas Eo, Er, and Elr exhibit a protracted maturation beyond 1 year of age. Similar to area 36c of the perirhinal cortex, area Ei of the entorhinal cortex exhibits a developmental profile in between those of rostral and caudal areas of the entorhinal cortex. Considering that area 36c shares substantial interconnections with the parahippocampal cortex and that area Ei shares structural and functional characteristics with both the rostral and caudal subdivisions of the entorhinal cortex, it is not surprising that the volumetric development of both areas 36c and Ei follows an intermediate profile.

#### 4.1.2 Layers

The distinct layers within each subdivision of the perirhinal and parahippocampal cortices exhibited different profiles of volumetric development. The superficial layers I, II, and III of areas 36r and 36c exhibited an earlier maturation than the deep layers V and VI. In contrast, the superficial and deep layers of areas TF and TH exhibited an early volumetric maturation between birth and 6 months of age. Accordingly, layers I and II develop early in most subdivisions of the entorhinal cortex (Piguet et al. 2020). Layer III exhibits an early maturation in Ec and Ecl, a two‐step/early maturation in Ei and a late maturation in Er. Layers V and VI exhibit an early maturation in Ec and Ecl, a two‐step early maturation in Ei, and a late maturation in Er. Thus, overall, the superficial layers of the perirhinal, parahippocampal, and entorhinal cortices, which constitute the first two stages in the cortical‐hippocampal loop of information processing toward the hippocampal formation (Lavenex and Amaral 2000), mature first. Interestingly, neurons in layer III of the entorhinal cortex send direct projections to the molecular layer of the subiculum and stratum lacunosum‐moleculare of CA1, and both of these layers mature earlier than the layers receiving intrahippocampal projections via the trisynaptic pathway including the dentate gyrus and CA3 (Jabès et al. 2011). In contrast, the deep layers of the entorhinal, perirhinal, and parahippocampal cortices mature relatively late, in particular in the rostral portion of the entorhinal cortex and the perirhinal cortex. Together, these findings suggest that cortical inputs can reach and be processed within hippocampal circuits relatively early, whereas hippocampal outputs might be directed mainly toward subcortical structures at early ages and only reach cortical areas at later stages of postnatal development.

### 4.2 Developmental Changes in Neuron Number

All layers of area 35 and layer III of area TF exhibited a decrease in neuron number between birth and adulthood, which was most prominent between birth and 6 months of age, suggesting that apoptosis (i.e., programmed cell death) occurs during early postnatal development in these areas. These findings are consistent with apoptosis reported in the cerebral cortex of several mammalian species during development (Causeret et al. 2018; Chan et al. 2002; Nikolic et al. 2013). Although this phenomenon seems to involve several cortical areas and various classes of neurons, the rate of apoptosis differs between cortical areas and some neuronal populations are simply refined while some transient populations of cells are completely eliminated (Blanquie et al. 2017; Causeret et al. 2018). In the rat perirhinal cortex, the number of Golgi‐stained neurons decreases during postnatal development (Furtak et al. 2007). However, the unclear mechanisms by which neurons become impregnated with Golgi staining makes those findings difficult to interpret because some changes in total cell numbers might reflect changes in the efficiency of the stain at different developmental stages (Furtak et al. 2007; Lavenex 2009).

In primates, apoptotic activity occurs in the cerebral cortex prenatally (Chan et al. 2002; Rakic and Zecevic 2000). However, it is unclear whether this phenomenon stops at birth or continues postnatally. Analyses of neuronal densities suggest that neuronal death may extend into the postnatal period (O'Kusky and Colonnier 1982; Rabinowicz et al. 1996), but density measures are not easy to interpret, especially as the volume of brain regions changes during development. Human studies using stereological methods reported a decrease in neuron number between birth and adulthood in some brain regions, such as the mediodorsal nucleus of the thalamus (Abitz et al. 2007), but no difference were found in the total number of neurons in the cerebral cortex (Larsen et al. 2006). Our study reports a postnatal decrease in the number of neurons in the nonhuman primate cortex and suggests that apoptosis occurs postnatally in discrete layers and cortical regions, which will not be detectable in a global analysis of the entire cortex. Indeed, the decrease in neuron numbers in the perirhinal and parahippocampal cortices mostly involved area 35 and layer III of area TF. The reasons why these regions, and especially area 35 exhibited a major decrease in neuron number is intriguing. Similar to the postnatal elimination and refinement of axonal connections and dendritic properties (Elston and Fujita 2014; Innocenti and Price 2005), postnatal apoptosis may contribute to the functional selection and refinement of cortical networks during development (Causeret et al. 2018). Consistent with an earlier development of the parahippocampal cortex as compared to the perirhinal cortex, the developmental decrease in neuron number occurred essentially between birth and 6 months in area TF while it occurred more gradually in area 35. Interestingly, axonal and dendritic pruning in sulci is thought to be important for sulcal and gyral development in childhood and adolescence, as it releases the tension exerted laterally and vertically, and allows changes in its curvature (White et al. 2010). Whether the postnatal decrease in neuron number in area 35 contributes to changes in the morphology of the rhinal sulcus remains to be investigated.

In parallel to the developmental decrease in the number of mature neurons in specific regions of the perirhinal and parahippocampal cortices, we observed a decrease in the number of immature neurons in the perirhinal cortex. Together with the amygdala and the rostral portion of the entorhinal cortex, layer II of the monkey perirhinal cortex contains a large population of immature neurons (Villard et al. 2023; Zhang et al. 2009). While previous studies reported clear evidence of structural and functional plasticity within the monkey medial temporal lobe following hippocampal damage (Chareyron et al. 2016; Lavenex et al. 2007b; Villard et al. 2023; Zhang et al. 2009), the fate of these immature neurons during normal postnatal development remains unclear. Here, we showed that the number of immature neurons in layer II of area 36 exhibited a two‐step decrease, between birth and 6 months of age, and between 1 year of age and adulthood. A similar decrease in the number of immature neurons between 1 year of age and adulthood has been documented in the monkey amygdala (Chareyron et al. 2012, 2021), a region sharing strong connections with the perirhinal cortex. Notably, immature neurons in the amygdala differentiate during adolescence in primates, thus increasing the mature neuron population in adulthood (Chareyron et al. 2012, 2021; Sorrells et al. 2019). Our findings in layer II of area 36 suggest a similar phenomenon, as the decrease in the number of immature neurons between 1 year of age and adulthood coincided with a subtle increase in the number of mature neurons, maintaining the overall neuronal count (including both immature and mature neurons) stable after 6 months of age. However, our results also suggest that in the perirhinal cortex, many of these immature neurons die between birth and 6 months of age, a period during which a proportion of mature neurons die as well. Interestingly, a recent study showed that the postnatal human temporal lobe contains many young neurons migrating into the entorhinal cortex and adjacent regions during the first 3 years of life (Nascimento et al. 2024), although this migratory stream was reportedly absent in monkeys. Altogether, these findings suggest a dynamic neuronal development in certain medial temporal lobe regions, with high levels of neuronal plasticity in late‐maturing regions containing immature neurons, including the amygdala, the perirhinal cortex and the rostral portion of the entorhinal cortex.

### 4.3 Developmental Changes in Neuronal Soma Size

The perirhinal and parahippocampal cortices exhibited changes in neuronal soma size during postnatal development. Consistent with previous findings in the entorhinal cortex (Piguet et al. 2020), neuronal soma size exhibited an increase between birth and 6 months of age, followed by a decrease into adulthood. Although some of these age‐related differences in neuronal soma size may be partially influenced by changes in neuron number in a few layers and areas (see above), this is clearly not the case in other areas where the number of neurons does not differ between ages.

Developmental changes in neuronal soma size differed between distinct layers and subdivisions, and reflected a clear rostrocaudal gradient. Areas TF and TH exhibited relatively fewer changes in neuronal soma size compared to areas 35 and 36r, while area 36c exhibited profiles in between those of area 36r and TF. This gradient was particularly evident in layer II, in which neurons exhibited a transient‐increase profile in areas 35 and 36r, an early increase profile in area 36c, and a very early maturation profile in areas TF and TH. Similarly in layer III, neuronal soma size increased transiently and decreased thereafter to reach adult size in most subdivisions, except in area TH of the parahippocampal cortex where neuronal soma size did not differ between ages. Interestingly, those neurons give rise to projections toward the superficial layers of the caudal entorhinal cortex (Suzuki and Amaral 1994b), which also exhibit few postnatal changes in neuronal soma size, particularly layer II of areas Ec and Ecl (Piguet et al. 2020). In contrast, the deep layers of the different subdivisions of the entorhinal, perirhinal, and parahippocampal cortices exhibited important postnatal changes in neuronal soma size. These results are consistent with the differential volumetric maturation described earlier, and further suggest that neurons located in the superficial layers of the parahippocampal cortex and the caudal portion of the entorhinal cortex exhibit the earliest maturation, whereas neurons located in the deep layers of the perirhinal cortex and the rostral portion of the entorhinal cortex exhibit the latest maturation.

The fact that distinct subdivisions and layers of the perirhinal and parahippocampal cortices exhibited different developmental profiles of neuronal soma size is consistent with studies reporting different developmental patterns of axonal, dendritic arborization, and spine formation across different cortical and subcortical regions of the primate brain (Elston and Fujita 2014; Elston et al. 2010, 2011; Huttenlocher and Dabholkar 1997; Innocenti and Price 2005; Oga et al. 2017, 1982; Rakic et al. 1986). In particular, the transient increase in neuronal soma size between birth and 6 months of age in the perirhinal and parahippocampal cortices (current study), as well as in the entorhinal cortex (Piguet et al., 2020), seems to concur with the synaptogenesis observed between birth and 4 months of age in monkeys (Elston et al. 2010, 2011; Oga et al. 2017; Rakic et al. 1986) and between birth and 15 months of age in humans (Huttenlocher and Dabholkar 1997). Subsequently, the decrease in soma size observed in the monkey entorhinal, perirhinal, and parahippocampal cortices between 6 months of age and adulthood seem to concur with synaptic pruning (Elston et al. 2010, 2011; Huttenlocher and Dabholkar 1997; Oga et al. 2017). Interestingly, morphological analyses of layer III and V pyramidal cells across monkey visual areas V1, V2, V4, TEO, TE, and prefrontal area 12 showed that the rate and magnitude of postnatal synaptic pruning differ between cortical areas and layers (Elston and Fujita 2014; Elston et al. 2010; Oga et al. 2017). Pyramidal cells in areas V1, V2, and V4 ultimately prune more spines than they initially had at birth, whereas cells in more rostral areas such as areas TEO, TE, and 12 ultimately prune fewer spines than they initially grow, eventually leading to higher spine densities in higher order cortical areas. Similar patterns were observed in the human auditory and prefrontal cortices (Huttenlocher and Dabholkar 1997). Consistent with the distinct developmental patterns of synaptic pruning, neuronal soma size increases as much as it subsequently decreases in the parahippocampal cortex, while it increases more than it subsequently decreases in the perirhinal cortex, leading to larger neuronal soma size in adulthood (Villard et al. 2024). By further analyzing neuronal soma area, dendritic branching and tree size in cortical areas in V1, V2, V4, TEO, TE, and 12, Elston et al. (2010) also observed different patterns of development across regions. Although these morphological aspects do not necessarily follow the same developmental patterns as spine density in those areas, associative areas tend to grow larger somas and dendritic territories while primary visual areas exhibit a decrease in both (Elston et al. 2010; Oga et al. 2017).

Interestingly, a study focusing specifically on layer III neurons of the monkey anterior ventral inferotemporal cortex—which corresponds to our rostral TE and area 36c—showed that the dendritic trees become larger from birth to 7 months of age and thereafter diminish in size to adulthood (Elston et al. 2011), thus consistent with the developmental changes in neuronal soma size that we observed in layer III of most subdivisions of the perirhinal and parahippocampal cortices. Together, these findings suggest that the developmental profiles of neuronal soma size in the perirhinal and parahippocampal cortices accompany distinct changes in neuronal morphology that reflect the functional differentiation of neurons and the complexification of neuronal circuits across brain hierarchies.

### 4.4 Development of the Medial Temporal Lobe Memory System

Here, we showed that the distinct subdivisions and layers of the monkey perirhinal and parahippocampal cortices exhibit different developmental profiles of structural development. Overall, the parahippocampal cortex exhibited an earlier maturation than the perirhinal cortex, with superficial layers exhibiting an earlier maturation than the deep layers. These developmental profiles parallel the profiles previously observed in the distinct subdivisions and layers of the monkey entorhinal cortex, with which they are interconnected (Piguet et al. 2020). The present work completes an important set of studies on the structural development of the monkey medial temporal lobe structures (Chareyron et al. 2012; Jabès et al. 2010, 2011; Piguet et al. 2020) and contributes to a better understanding of the structural and functional maturation of the distinct functional circuits within the primate medial temporal lobe memory system. We previously proposed that the differential maturation of distinct entorhinal–hippocampal circuits underlies the differential emergence of “hippocampus‐dependent” memory processes during the first years of life (Lavenex et al. 2024; Lavenex and Banta Lavenex 2013). We will now further evaluate this theory by discussing the maturation of the distinct functional circuits to which the perirhinal and parahippocampal cortices contribute.

#### 4.4.1 Early Maturation of the Parahippocampal Cortex

The monkey parahippocampal cortex receives the vast majority of its cortical inputs from visuospatial areas V4, caudal TEO, the retrosplenial and the posterior parietal cortices, and constitutes the first stage in the integration of visuospatial information within the medial temporal lobe memory system (Lavenex and Amaral 2000; Lavenex et al. 2024; Suzuki and Amaral 1994a). Accordingly, the parahippocampal cortex has been linked to the processing of spatial and contextual information, particularly the processing of spatial scenes (Aguirre et al. 1996; Lee et al. 2008; Mormann et al. 2017; Rolls 2023; Rolls et al. 1997).

In contrast to the perirhinal cortex, the parahippocampal cortex exhibited an early volumetric maturation between birth and 6 months of age. These changes were accompanied by a transient increase in neuronal soma size at 6 months, and a decrease in neuron number in layer III of area TF essentially between birth and 6 months. Despite subtle differences, areas TF and TH exhibited similar profiles of volumetric and neuronal soma size development. Of all regions analyzed, the ones exhibiting the earliest maturation were the superficial layers of the parahippocampal cortex. Although the volume of these layers continued to increase between birth and 6 months of age, neurons in layer II and III already exhibited an adult soma size at birth and did not exhibit major differences between age groups. These neurons give rise to feedforward projections to the superficial layers of the caudal subdivisions of the entorhinal cortex (Suzuki and Amaral 1994b), which in turn give rise to distinct projections toward other hippocampal structures (Amaral et al. 2024; Witter et al. 1989). Accordingly, neurons in layer II of the caudal entorhinal cortex (Ec, Ecl) do not exhibit postnatal changes in soma size, and layer III neurons exhibited larger somas at birth than in adulthood (Piguet et al. 2020). The early maturation of neurons in the superficial layers of the parahippocampal cortex may have thus influenced the maturation of entorhinal neurons to which they project. Similarly, the subiculum, presubiculum and parasubiculum—which are strongly interconnected with the caudal entorhinal cortex—exhibit a very early developmental profile both in terms of volume and neuronal soma size (Jabès et al. 2010). Note that the parahippocampal cortex also sends projections to the subiculum and the presubiculum (Van Hoesen et al. 1979), further supporting the idea of a synchronized maturation of interconnected medial temporal lobe structures. Consistent with the early functional maturation of head‐direction cells in the presubiculum and parasubiculum (Langston et al. 2010), Piguet et al. (2020) proposed that the very early development of the caudal entorhinal cortex and the subicular complex may underlie the emergence of path integration abilities in children between 6 and 12 months of age (Acredolo 1978; Bremner et al. 1994; Rieser and Heiman 1982). Although there is no evidence for a direct contribution of the parahippocampal cortex to path integration, as this region has been primarily associated with allocentric spatial memory, spatial view cells found in the parahippocampal cortex (Rolls 2023) could contribute to recalibrating head‐direction cells during path integration (Knierim et al. 1995; Taube 2007; Taube and Burton 1995). Our findings show that the main route conveying visuospatial information to the entorhinal cortex and hippocampal structures is relatively structurally mature in the first postnatal months.

The overall volumetric maturation of the parahippocampal cortex between birth and 6 months of age parallels the maturation of the caudal entorhinal cortex and area CA1 of the hippocampus (Jabès et al. 2011; Piguet et al. 2020). Together with the subicular complex, these regions contain several types of cells involved in spatial information processing, which exhibit different developmental profiles (Langston et al. 2010; Taube 2007). The parahippocampal cortex contains spatial view cells which encode visuospatial information based on where an individual looks in the environment, regardless of its physical position (Rolls 2023; Rolls et al. 1997), and thus plays an important role in building and remembering allocentric representations of the environment based on visual inputs. Although the functional maturation of these cells has not yet been explored in primates, the early maturation of parahippocampal–entorhinal–CA1 connections between birth and 6 months of age in monkeys is consistent with our earlier model proposing that this functional circuit underlies the emergence of basic allocentric spatial processing reliably observed in children at 2 years of age (Lavenex et al. 2024; Lavenex and Banta Lavenex 2013; Newcombe et al. 1998; Ribordy et al. 2013; Ribordy Lambert et al. 2017). The maturation of feedback projections via the deep layers of the caudal entorhinal cortex and parahippocampal cortex to other cortical areas may also mark the beginning of long‐term memory consolidation in these areas, which is concordant with the offset of infantile amnesia in 2‐year‐old children (Lavenex et al. 2024; Lavenex and Banta Lavenex 2013).

#### 4.4.2 Late Maturation of the Perirhinal Cortex

The monkey perirhinal cortex receives most of its cortical inputs from visual areas TE and rostral TEO, as well as substantial inputs from the amygdala (Stefanacci et al. 1996; Suzuki and Amaral 1994a). Within the medial temporal lobe memory system, the perirhinal cortex thus constitutes the first stage in the integration of visual object information (Lavenex and Amaral 2000; Suzuki and Amaral 1994a). Accordingly, the perirhinal cortex has been consistently linked to visual object perception, association, and discrimination (Buckley and Gaffan 1998; Fujimichi et al. 2010; Miyashita 2019; Naya et al. 2003; Suzuki and Naya 2014). Moreover, amygdala activity facilitates the transmission of inputs from the perirhinal cortex to the entorhinal cortex, suggesting a contribution of the perirhinal cortex to the emotional enhancement of memory (Paz et al. 2006).

In contrast to the parahippocampal cortex, the perirhinal cortex exhibited a protracted volumetric maturation continuing beyond the first postnatal year, accompanied by a transient increase in neuronal soma size resulting in larger neurons in adulthood than at birth. Intriguingly, area 35 was volumetrically mature at birth, but exhibited a gradual decrease in neuron number from birth to adulthood. The superficial layers I–III of areas 36r and 36c, which give rise to feedforward projections to the rostral portion of the entorhinal cortex, exhibited a relatively earlier volumetric maturation than the deep layers V–VI, which themselves give rise to feedback projections to other cortical areas. Similarly, the superficial layers I–III of areas Eo, Er, and Elr of the entorhinal cortex, which in turn give rise to distinct projections toward hippocampal structures, exhibited a relatively earlier volumetric maturation than the deep layers V–VI (Piguet et al. 2020). These findings suggest that, similar to what was observed for the parahippocampal cortex and the caudal portion of the entorhinal cortex, maturational processes of the rostral portion of the entorhinal cortex may partially depend on cortical inputs via the perirhinal cortex and thus follow the sequence of information processing in the medial temporal lobe.

The overall late structural development of the perirhinal cortex after 1 year of age is concomitant with the late development of the rostral entorhinal cortex, the dentate gyrus and area CA3 of the hippocampus (Jabès et al. 2010, 2011; Piguet et al. 2020). Consistent with our earlier model linking the protracted development of the dentate gyrus, area CA3 and rostral entorhinal cortex with the age‐related improvements in pattern separation, which subserve high‐resolution allocentric spatial representations (Lavenex et al. 2024; Lavenex and Banta Lavenex 2013), the perirhinal cortex may contribute to increase the precision of spatial representations by integrating information about individual objects. Interestingly, area 36c exhibited profiles of volumetric and neuronal soma size development in between those of the parahippocampal cortex and the rest of the perirhinal cortex. Compared to the rest of the perirhinal cortex, area 36c is more strongly connected to the parahippocampal cortex (Lavenex et al. 2004) and more active during spatial exploration, particularly its superficial layers (Chareyron et al. 2017). The rostrocaudal gradient of maturation of the perirhinal cortex suggests that the increase in the precision of spatial memories develops progressively after the emergence of the ability to form a basic allocentric spatial representation, which is dependent on the maturation of the parahippocampal cortex and caudal entorhinal cortex.

It was previously proposed that the immaturity of the dentate gyrus may underlie the phenomenon of childhood amnesia (Jabès et al. 2010; Josselyn and Frankland 2012), and that the gradual maturation of the trisynaptic hippocampal pathway subserves the gradual improvement, from 2 to 7 years of age, in our ability to create autobiographical memories that can be recalled later in life (Lavenex et al. 2024; Lavenex and Banta Lavenex 2013; Ribordy et al. 2013; Ribordy Lambert et al. 2017, 2015). Furthermore, the late maturation of the rostral entorhinal cortex has been proposed to contribute to the gradual improvement of temporal discrimination between events (Piguet et al. 2020). The late maturation of the perirhinal cortex may contribute to the gradual improvement of object and concept discrimination, providing the entorhinal cortex and hippocampus with more detailed experiential content to disambiguate events happening in similar contexts. Recent evidence suggests that the perirhinal cortex interacts with the dentate gyrus to subserve memories requiring the discrimination of similar objects (Miranda et al. 2021). Interestingly, both of these regions, together with the rostral entorhinal cortex and amygdala contain immature neurons which, in the dentate gyrus, have been proposed to facilitate the distinction of overlapping memories (Aimone et al. 2009; Sahay et al. 2011). Whether the pool of immature neurons that gradually decreases postnatally in the perirhinal cortex may play a complementary role to disambiguate similar objects or emotional memories remains to be determined.

## 5 Conclusion

The aim of this study was to characterize the postnatal structural development of the monkey perirhinal and parahippocampal cortices, by providing estimates of volume, neuron number, and neuronal soma size for each subdivision and layer at four postnatal ages. Consistent with the differential maturation of the distinct regions of the hippocampal formation (Jabès et al. 2011), including the entorhinal cortex (Piguet et al. 2020), our results showed that the distinct layers and subdivisions of the perirhinal and parahippocampal cortices exhibit different postnatal developmental profiles. Overall, the parahippocampal cortex exhibits an early volumetric maturation between birth and 6 months of age, while the perirhinal cortex exhibits a protracted maturation continuing beyond the first postnatal year. The changes in volumes and neuronal soma size also indicated that superficial layers tend to mature earlier than the deep layers, especially in rostral subdivisions. In some regions, and particularly area 35, these changes were accompanied by a decrease in neuron number, further suggesting that apoptosis occurs during postnatal development. These findings parallel those showing the differential maturation of the rostral and caudal entorhinal cortex, which are interconnected with the perirhinal and parahippocampal cortices, respectively. Altogether, they support the theory that the differential maturation of distinct hippocampal circuits underlies the emergence of specific “hippocampus‐dependent” memory processes.

## Author Contributions

Justine Villard contributed to study design, data collection, analysis and interpretation, writing of the manuscript; Loïc J. Chareyron contributed to data analysis and interpretation, writing of the manuscript; Pamela Banta Lavenex and David G. Amaral contributed to study design, data interpretation and writing of the manuscript. Pierre Lavenex contributed to study design, data analysis and interpretation, writing of the manuscript, overall project supervision.

## Funding

This work was supported by Swiss National Science Foundation Grants P00A‐106701, PP00P3‐124536, and 310030_143956; US National Institutes of Health Grants MH041479 and NS16980; and California National Primate Research Center Grant OD011107.

## Conflicts of Interest

The authors declare no conflicts of interest.

## Data Availability

The data that support the findings of this study are available from the corresponding author upon reasonable request.
